# Effects of Community Participation on Improving Uptake of Skilled Care for Maternal and Newborn Health: A Systematic Review

**DOI:** 10.1371/journal.pone.0055012

**Published:** 2013-02-04

**Authors:** Cicely Marston, Alicia Renedo, C. R. McGowan, Anayda Portela

**Affiliations:** 1 Senior Lecturer, Department of Social and Environmental Health Research, London School of Hygiene and Tropical Medicine, London, United Kingdom; 2 Research Fellow, Department of Social and Environmental Health Research, London School of Hygiene and Tropical Medicine, London, United Kingdom; 3 Doctoral Student, Department of Global Health and Development, London School of Hygiene and Tropical Medicine, London, United Kingdom; 4 Technical Officer, Department of Maternal, Newborn, Child and Adolescent Health, World Health Organization, Geneva, Switzerland; Indiana University and Moi University, United States of America

## Abstract

**Background:**

Despite a broad consensus that communities should be actively involved in improving their own health, evidence for the effect of community participation on specific health outcomes is limited. We examine the effectiveness of community participation interventions in maternal and newborn health, asking: did participation improve outcomes? We also look at how the impact of community participation has been assessed, particularly through randomised controlled trials, and make recommendations for future research. We highlight the importance of qualitative investigation, suggesting key areas for qualitative data reporting alongside quantitative work.

**Methods and findings:**

Systematic review of published and ‘grey’ literature from 1990. We searched 11 databases, and followed up secondary references. Main outcome measures were the use of skilled care before/during/after birth and maternal/newborn mortality/morbidity. We included qualitative and quantitative studies from any country, and used a community participation theoretical framework to analyse the data. We found 10 interventions. Community participation had largely positive impacts on maternal/newborn health as part of a package of interventions, although not necessarily on uptake of skilled care. Interventions improving mortality or use of skilled care raised awareness, encouraged dialogue and involved communities in designing solutions–but so did those showing no effect.

**Discussion:**

There are few high-quality, quantitative studies. We also lack information about why participation interventions do/do not succeed – an area of obvious interest for programme designers. Qualitative investigation can help fill this information gap and should be at the heart of future quantitative research examining participation interventions – in maternal/newborn health, and more widely. This review illustrates the need for qualitative investigation alongside RCTs and other quantitative studies to understand complex interventions in context, describe predicted and unforeseen impacts, assess potential for generalisability, and capture the less easily measurable social/political effects of encouraging participation.

## Introduction

There has been a broad consensus that communities should be actively involved in improving their own health [1]–[4]. Yet evidence for the effect of community participation – here broadly defined as members of a community getting involved in planning, designing, implementing, and/or adapting strategies and interventions [5] – on specific health outcomes is limited.

The rationale for community participation in health programmes has included responding better to communities’ needs, designing programmes that account for contextual influences on health (such as the effects of local knowledge or cultural practices), increasing public accountability for health, and it being a desirable end in itself [4], [6], [7]. Involving communities is thought to be crucial in improving health equity, healthcare service delivery and uptake [8], and has been repeatedly recommended in international conferences and charters [1]–[4].

Despite the apparent consensus about the value of participation, there is no single agreed concept of what participation is or should be [9]–[14] and programmes often develop without an explicit definition [12]. In 1991, a World Health Organization Study Group defined community involvement in health as: “a process whereby people, both individually and in groups, exercise their right to play an active and direct role in the development of appropriate health services, in ensuring the conditions for sustained better health and in supporting the empowerment of community to help development. [Community involvement in health] actively promotes people’s involvement and encourages them to take an interest in, to contribute to and take some responsibility for the provision of services to promote health” ([15] p. 10).

Participation approaches can be understood in terms of two broad categories [16]–[18]. The first is utilitarian [18], where participation is a discrete, short-term intervention [8], [16], [17], [19] and might involve for instance, “[using] community resources (land, labour and money) to offset the costs of providing services” ([18] p. 221). This approach has been criticised for treating participation as an add on or input to healthcare programmes [12], [20], and for ignoring the underlying context and processes contributing to communities’ health inequalities [10], [19]. It echoes the ‘medical’ and ‘health services’ models of community participation [12] where health is equated with absence of disease, and considered to be best achieved using biomedical approaches and delivery of high quality health services. Such programmes might for instance seek only to transfer technical information and skills.

The second approach aims to effect wider social and political transformation through social processes e.g. dialogue that develops over time [10], [16]–[18], [21] It focuses on lack of resources and social injustice as causes of poor health [12], [19] and sees community participation as a way to distribute power more evenly within and between communities, healthcare professionals, and the state, while also developing individuals’ and groups’ own abilities to participate in the process of change – improving their own health directly, or via community development activities [16], [17], [22]. In other words, this ‘community development’ or ‘empowerment’ approach sees participation as a longer-term process in which communities are actively involved in deciding on and implementing strategies to alter the socio-political, economic, and psychological conditions that shape their health [12], [19].

While this distinction between approaches is useful to help conceptualise types of participation intervention, in practice, elements of both approaches may exist within the same programme [10].

If community participation is viewed as a process of empowerment and a social practice it must necessarily be configured according to the social and political context, and change as the context changes [6], [10], [15], [19], [20]. Even the process of participation itself may affect health by developing community networks which in turn can provide social support, one effect of which might be to encourage healthy behaviours [23]. Viewing participation as a dynamic process rather than a discrete intervention implies that as well as looking at outcomes, evaluation should also account for intrinsic complexities such as the different forms participation can take in different settings, and the sustainability of participation over time – for instance, is the idea of participation accepted within the community, or is it temporarily tolerated while donors provide money for interventions [8]? The processes through which participation leads to change “might have some universal characteristics but the solution itself will be local” ([19] p. 89).

Interventions addressing what happens in the home, in families and in communities are crucial to improve maternal and newborn health. The availability of good quality services will not produce the desired health outcomes if individuals, families, and communities cannot make healthy decisions and act on them [24]. Problems that might need to be addressed include families and communities not providing the support women need (for instance, leaving women with an inadequate share of household food); women and families not recognising danger signs in pregnancy and childbirth; women being unwilling to use antenatal or childbirth services with skilled attendants; or access to appropriate services being limited by lack of transport or excessive costs. Community participation interventions may tackle these by encouraging communities themselves to identify problems, understand their root causes (e.g. barriers to timely referral to safer motherhood services) and mobilise necessary resources [25], as well as demanding their rights to health and high quality health services [26]. Community assessment of problems both raises awareness and stimulates social support and participation in problem-solving [24].

In this systematic review we examine the available evidence of the effectiveness of community participation interventions on maternal and newborn health, particularly on the uptake of skilled care during pregnancy, childbirth and after birth. We consider data from any population, where a community participation intervention was compared with no community participation. We include a range of measures of maternal and newborn health (see below). We include experimental and quasi-experimental quantitative studies, and qualitative studies.

We identify limitations in the quantity, scope, reporting, and design of previous studies. We then discuss how we can improve future research – including randomised controlled trials (RCTs), which are increasingly used to test complex interventions – to understand the impact of involving communities, both in maternal and newborn health and more widely. In particular, we highlight the importance of qualitative data and suggest key areas for qualitative investigation alongside an RCT or other quantitative work.

## Methods

### Criteria for Selecting Studies for this Review

This review focuses on improving uptake of skilled care for maternal and newborn health – a subject of particular interest to the World Health Organization, and is part of its wider efforts to improve the evidence base in this area. We considered data from any population. We reviewed published and ‘grey’ literature, including peer-reviewed journal articles, books, book chapters, electronic articles, reports, and theses.

We included studies:

- Published in English on or after 1 January 1990,
- Containing original, empirical data to examine effectiveness where community participation was implemented to improve maternal and newborn health. To avoid limiting our search unduly, we did not specify a particular definition of ‘community’.

We included outcome measures of uptake of skilled care during pregnancy, childbirth and after birth (for mother and newborn in the 28 days after the birth) as well as any direct measures of maternal and newborn health such as maternal mortality, maternal morbidity, or neonatal mortality.

We excluded:

- Studies of health personnel delivering services in the community that were previously clinic-based (i.e. where the only change was in the location of the service), and similarly, studies that only considered community health workers providing services in the community (these do not fit our definition of ‘community participation’ (see above)),
- Quantitative studies comparing the same population before and after an intervention. Secular changes in maternal and newborn health indicators mean that it is difficult to have confidence in causality attributed to the intervention in this type of study.

### Search Strategy and Selection Process

**Figure 1:**
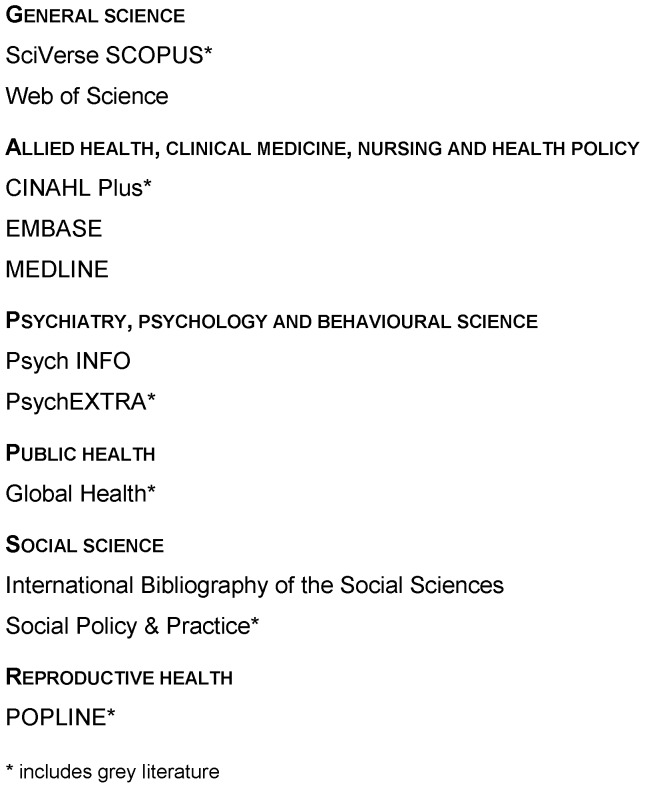
Databases used in the searches.

Our search strategy (Table 1) used a wide variety of search terms to produce a high sensitivity (and low precision) search. We searched 11 major databases from 12–18 March 2012 (Figure 1). We examined reference lists from relevant literature (e.g. included papers, reviews) for additional sources, and retrieved all items citing a key article [27] (N = 251).

**Table 1:** Search terms.

Focus of search terms	Search terms
**Maternal terms**	Abortion* OR Antenatal OR Birth* OR Childb* OR Delivery care OR Eclampsia OR Institutional delivery OR Intrapartum OR Matern* OR Midwi* OR Motherhood OR MTCT OR Obstetric care OR PMTCT OR Parturition OR Prenatal OR Peri-natal OR Post-partum OR Post partum OR Post-delivery care OR Post delivery care OR Pregnan* OR Puerperium care OR Reproductive health OR Stillbirth* OR Safe delivery OR (Skilled ADJ2 attendant)
or	
**Newborn terms**	Birth asphyxia OR Breastfe* OR Community midwi* OR Fetal nutrition disorder* OR Foetal nutrition disorder* OR Hypoxic ischemic encephalopathy OR Hypoxic ischaemic encephalopathy OR Infant OR Intra-partum OR Intra partum OR Integrated management of childhood illness OR IMCI OR Newborn* OR Neonat* OR Perinat* OR Post-natal OR Post natal OR TBA OR Traditional birth attendant
and	
**Community participation terms**	Collective action OR Collective mobili?ation OR Community Action OR Community mobili?ation OR Community capacity-building OR Community capacity building OR Community collaborat* OR Community conscienti?ation OR Community engagement OR Community intervention OR Community mobili?ation OR Community outreach OR Community involvement OR Community participation OR Community health program* OR Community initiative* OR Community-based health programme* OR Community-based intervention* OR Empower* OR Health Promotion OR Maximi?ing access OR Participatory intervention* OR Participatory approach* OR Social mobili?ation OR Social movement OR Social capital OR Social participation OR Village health worker* OR Women* group* OR (Community health ADJ2 program*) OR (Reduc* ADJ2 barriers to access)

AR scanned all titles/abstracts and discarded the clearly irrelevant ones. CM and AR together then narrowed the selection to all that were relevant, or where relevance was unclear: full texts were obtained for all these. CM and AR read the full texts, assessed them both for inclusion, and then, if included, for risk of bias at the study level using various guides, including those provided in the Cochrane Handbook [28]. Differences were resolved by discussion, with AP providing further input where necessary.

### Data Extraction

**Figure 2:**
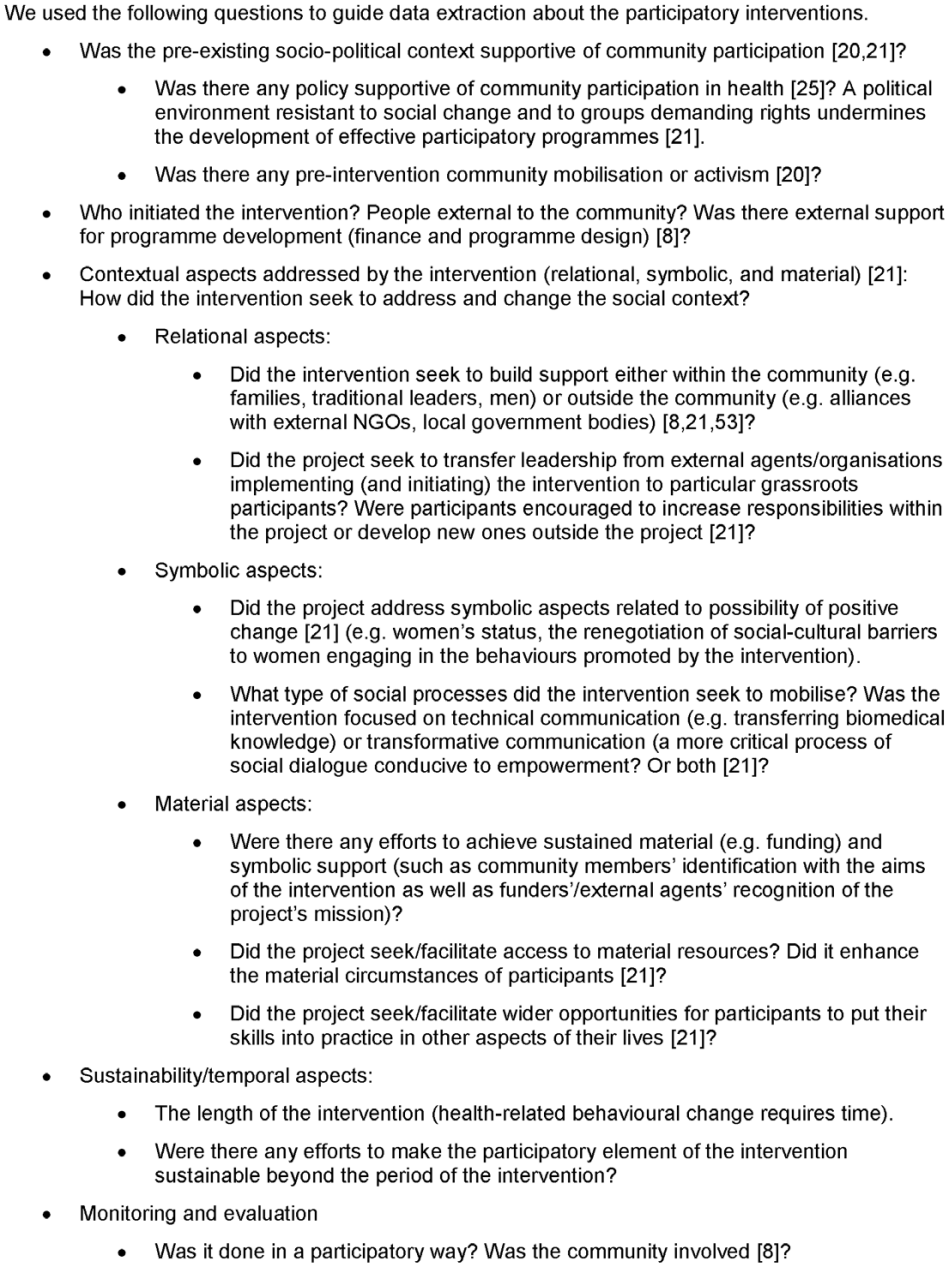
Elements of interest for community participation interventions.

We extracted data on study design and findings. Because we view participation as a process which can vary according to social context [8], [25], we critically appraised the studies with respect to their participation-related data, looking at elements of the process and context of participation suggested as promoting health improvement [8], [21] (more information in Figure 2). We did not extract data from studies judged to have high (as opposed to moderate or low) risk of bias, but provide their citations (Table S1). We did not consider a meta-analysis appropriate for these data: the studies describe a mix of interventions and contexts, and there is a lack of information on what was done in each location. We judged that combining odds ratios would risk masking differences in outcomes which might have arisen from differing contextual factors.

## Results

We screened 9,854 items, including 227 full texts (Checklist S1). Fifteen texts met our inclusion criteria, pertaining to 10 separate interventions: seven with low or moderate risk of bias, and three with high risk of bias (the latter are not considered further here).

In the included studies, community participation interventions involved encouraging people to think and talk about their health problems and services, and acting, or helping them act, on what they said. We extracted data from each study about elements of participation that have been suggested as important for health improvement but although studies referred to some of these elements, there was not enough consistent detail to allow a detailed analysis (we provide specific detail for information in the appendix – Table S1).

Most of the included studies were quantitative: only one primarily qualitative study is included in the final selection [29], largely because most qualitative studies contained no information – even brief remarks – about our outcomes of interest.

Several interventions were based on the innovative Warmi project in Bolivia, which aimed to improve maternal and child health using facilitated women’s groups [30]. The groups used “autodiagnosis” (similar to participatory action research [31], [32]) to identify and prioritise local problems, develop action plans accordingly, implement those plans, and then evaluate their own efforts [30], [33]. All groups identified the need to increase knowledge of reproduction, contraceptive use, and danger signs in pregnancy; improve immediate newborn care; and increase the proportion of women receiving skilled childbirth care [30]. Actions taken included participatory development of education materials [34], savings schemes, and literacy programmes [33].

### Impact of Community Participation Interventions

Five of the included interventions were associated with positive effects on maternal and neonatal outcomes:

- In Makwanpur, Nepal, a women’s group intervention, based on the Warmi project [30], assessed using a cluster RCT (cRCT), had a positive effect on antenatal care use (adjusted odds ratio (AOR) 2.82, 95% confidence interval 1.41–5.62) and, compared with women in control areas, women in the intervention areas were more likely to have given birth in a health facility or with a trained attendant (AOR = 3.53, 1.54–8.10). There were improvements in visiting facilities for skilled care in the event of maternal (AOR = 3.37, 1.78–6.37) or infant (AOR = 2.84, 1.65–4.88) illness. Improvements in care practices by traditional attendants during births at home e.g. use of clean childbirth kits (AOR = 4.59, 2.83–7.45), birth attendant washing hands (AOR = 5.50, 2.40–12.60), and use of boiled blades to cut the umbilical cord (AOR = 3.47, 1.39–8.69) were also reported. Neonatal mortality rates were lower in the intervention areas (AOR = 0.70, 0.53–0.94) as were maternal mortality ratios (AOR = 0.22, 0.05–0.90). Stillbirth and maternal morbidity did not differ between the two groups [27], [33], [35], [36]. Health services were strengthened in both intervention and control areas. See Tables 2 and S1 for further details about this and the other included interventions.
- In India, a very similar women’s group intervention – based on the Warmi project and also assessed using a cRCT – did not improve skilled-care seeking behaviour, but improved neonatal mortality (AOR = 0.68, 0.59–0.78) and care practices by traditional attendants during births at home e.g. use of safe childbirth kits [37], [38].
- A quasi-experimental study of a participatory young people’s reproductive health promotion project in Nepal showed mixed results. The study compared one urban and one rural site where interventions had been designed with community participation – “using an action planning process in which needs assessment results were shared and analysed with the community, and community task forces were created to set priorities and design feasible interventions” ([39] p. 215) – with one urban and one rural control site where there had been no participation. The authors claim the intervention was linked to a reduction in urban/rural differentials in use of antenatal care or birth in a health facility, but they also reported that overall use of antenatal facilities decreased. The authors report that the participatory approach had empowered young people (by, for instance, their learning to negotiate with village development committee, and feeling able to demand government funds to continue the project), and increased provider accountability and community demand for information [39], [40].
- In Kenya, health effects of joint decision making through dialogue between community members and service providers were examined in 12 areas (across six districts) compared with 12 matched control areas. There were improvements in a number of indicators, including childbirth in a health facility (41% in intervention sites vs. 23% in control sites, p = 0.000). The authors also report improved accountability of service providers to the communities they served [41].
- Maternal death audits in India [29] involved interviewing people connected with women who had died to try to understand what had gone wrong. The process drew attention to errors that had led to deaths, which were then presented to the communities, making “invisible problems visible” ([29] p. 75). This led to a response to tackle these from both community members and service providers and, the study reports, improved accountability of providers. Few quantitative data are given in the primarily qualitative report about this intervention but in at least one area it records a rise in proportions of women giving birth in facilities from 23% to 39% (although there was no comparison area).

**Table 2:** Summary of findings and when community participation occurred in the included studies.

Intervention (briefidentifier, citations)	Relevant improvements shown?	Community help identify problems?§			Community participation in…		
		*Community* *member/health* *provider dialogue/* *consultation?*	*Participatory* *identification* *of local* *problems?*	*Using or creating data to prompt discussion of local needs?*	*Design of interventions?*	*Implementing interventions?*	*Monitoring and evaluation?*
Bangladesh: 3 districts,women’s groups. *Azad et al.* †	No	Perhaps**	Yes	No**	Yes	Yes	Yes
Malawi (MaiMwana) women’sgroups. *Lewycka et al.;* *Rosato etal.* †	No	Perhaps**	Yes	No**	Yes	Yes	Yes
Nepal, Makwanpur women’sgroups. *Manandhar et al.;* *Morrison et al.; Wade etal* †	Yes. Compared with control clusters, intervention clustershad lower: neonatal mortality rates (Adj OR: 0.70, 95%CI:0.53–0.94), and maternal mortality ratio (Adj OR: 0.22,0.05–0.90); they had more ANC use (Adj OR: 2.82,1.41–5.62), births in facilities (Adj OR: 3.53, 1.54–8.10)and visits to facilities for maternal (Adj OR: 3.37, 1.78–6.37)or infant (Adj OR: 2.84, 1.65–4.88) illness.	Yes	Yes	No**	Yes	Yes	Somewhat
India: Ekjut. Jharkhand andOrissa women’s groups.*Tripathy et al.; Rath et al.* †	Yes. Neonatal mortality rate was lower in intervention thancontrol clusters (Adj OR: 0.68, 0.59–0.78).	Yes	Yes	No**	Yes	Yes	Yes
Kenya. *Kaseje etal.* ‡	Yes. More births in facilities in intervention than controlsites (41% vs. 23%, p = 0.000)	Yes	Within health services	Yes	Suggestedimprovements	No	Somewhat
Nepal young people.*Malhotra et al.; Mathur et al.* ‡	Marginal. Authors claim intervention reduced differentialsin youth reproductive health outcomes but results are mixedand interpretation difficult.	Perhaps**	Yes	Yes	Yes	Demandservices (p. 231)	Authors say yes but do not provide detail
Mapedir maternal deathaudits. *UNICEF*	Yes.* More community awareness/actions toprevent maternal death. Orissa: increased childbirth ininstitutions 23% to 39% (NB: no control group).	Indirectly	Yes (in audit process)	Yes	Yes (post-audit)	Yes (post-audit)	n/a

† Low risk of bias;

‡ Moderate risk of bias;

* Mostly relying on qualitative data;

** Not stated explicitly;

§ Categories for when in the intervention participation occurred are based on commonly-used approaches in participatory action research (e.g. see Baum F, MacDougall C, Smith D (2006) Participatory action research. J Epidemiol Community Health 60∶ 854–857).

Two of the included interventions showed no impact. Both were women’s group interventions based on the Warmi project. They were conducted in Bangladesh [42] and Malawi [43], [44] and assessed using cRCTs. There was no significant impact of the intervention on the key maternal or newborn outcomes.

### Findings from Studies which were not Included

Two further community participation programmes increased births in health facilities [45], [46] and one additional programme also reduced neonatal mortality and stillbirths [47], [48] but unlike the included studies, their success could not be attributed solely to the participation component. This was because the studies compared no intervention at all with participation plus quality of care improvements [45], [46] or participation plus community health worker training [47], [48] – in other words the participation element was not isolated.

One study compared the effects of introducing village-based community nurse services versus a participation intervention (village health committees plus community health volunteers) alone, or nurse services and participation combined [49]. Unfortunately they do not report on newborn outcomes directly, although they found that infant mortality was *higher* in the two participation areas (where it was introduced alone and also where it was combined with the nurse services), and only reduced in the areas with nurse services alone, suggesting that, in this case, the health committees and community volunteers may even have had a detrimental effect.

### Participation in the Successful and Unsuccessful Interventions

Here we summarise some of the characteristics of the participation interventions that were reported.

The successful interventions – those that resulted in positive maternal and neonatal outcomes – all involved raising community awareness of maternal and newborn health problems, and encouraging dialogue, which some claim is a precondition for behaviour change [6], as opposed to simply providing information.

Where problems were identified, communities were often involved in designing and sometimes implementing solutions. For example, establishing community-generated funds for maternal and infant care [27], or improving or providing transport for cases of obstetric emergency [29], often using local resources (e.g. existing vehicles) [29].

In Kenya, dialogue between community members and health service providers was a core characteristic of the intervention [41], with actions for improvement agreed between them. In India, community generation of data in maternal death audits drew attention to clusters of deaths in certain geographical areas, and prompted community and health provider responses [29].

However, the two interventions with no impact on maternal and newborn health outcomes [42], [43] were also initiated externally, raised awareness, encouraged dialogue, and involved communities in designing solutions. It is particularly interesting to consider that these were two of four very similar interventions based on the Warmi project [30] and assessed using RCTs – the four quantitative studies in this review with the least risk of bias. All four were modelled on the same original project, employed the same women’s group approach, and some researchers were involved in all four projects. Yet two (in Nepal and India) were successful, and two (in Bangladesh and Malawi) unsuccessful (in that they did not record any effects of community participation on the outcomes of interest). For Bangladesh the authors speculate that this may have been because of lower coverage of women’s groups compared with the successful Nepal intervention, differences in local context, and differences in “quality of the intervention” ([42] p. 1200) – but do not report any detailed investigation of these factors [42].

Table 2 summarises key outcomes of the included studies and also – in the absence of detailed data about how activities were carried out – summarises when in the included interventions participation occurred.

## Discussion

The included studies suggest community participation has largely positive impacts on maternal and newborn health as part of a package of interventions, although not necessarily on uptake of skilled care. The limitations of the data prevent us from drawing firm conclusions about what characterises successful participation interventions. Below we describe these limitations, and go on to discuss the broader challenges faced in designing future studies.

This systematic review reveals the need for better studies of community participation in maternal and newborn health. We found very few quantitative studies that included basic features to reduce risk of bias, such as comparison groups or randomisation. As has been found in other areas of health interventions research [50], little information was available to help understand why participation interventions worked or did not work. There was also a lack of information on sustainability or costs (see Table S1). To understand why participation appears to have improved health in some contexts and not others, it would be useful to know not only whether but also how certain activities had been carried out. For instance, “dialogue” between health service providers and community members may have been reported in most of the studies, but how was it conducted? Who was able to engage in dialogue? Were some groups excluded? Was dialogue open? Was it respectful?

Future studies should collect and publish qualitative data, ideally using common areas of reporting to explain why participation might or might not have improved health. The fact that seemingly similar interventions were successful in some locations [27], [38] but not others [42], [43] underlines the importance of such data. For instance, in Bangladesh, the authors point to contextual factors to explain the relatively unsuccessful outcomes compared with a very similar intervention in Nepal. Qualitative investigation alongside the RCT would allow us to understand what the key contextual differences were and how these contributed to success or failure. It is difficult, and perhaps undesirable, to standardise community participation interventions because of the need to be sensitive to context, and qualitative data would also allow programmes to assess which elements of any given approach – such as emphasis on specific types of dialogue – could be implemented elsewhere. Even where qualitative work has been carried out as part of large trials, very little analysis has been published.

Qualitative work will also capture wider benefits from participation not easily measured in quantitative studies. Although the trials have been good at measuring quantifiable effects, we also need to examine the wider social change (such as change in women’s status) that is a key rationale for participatory interventions [25], [51], and can lead to sustained health improvements. For instance, qualitative work revealed how the maternal death audits intervention in India provoked a response from both community members and health providers [29]. This illustrates a point raised in the literature: that interventions may build ‘community voice’ (i.e. a community’s capacity to articulate and assert its needs) and persuade people in power to respond to community demands [21]. If this does indeed happen, it should be captured along with more easily quantifiable outcomes.

**Figure 3:**
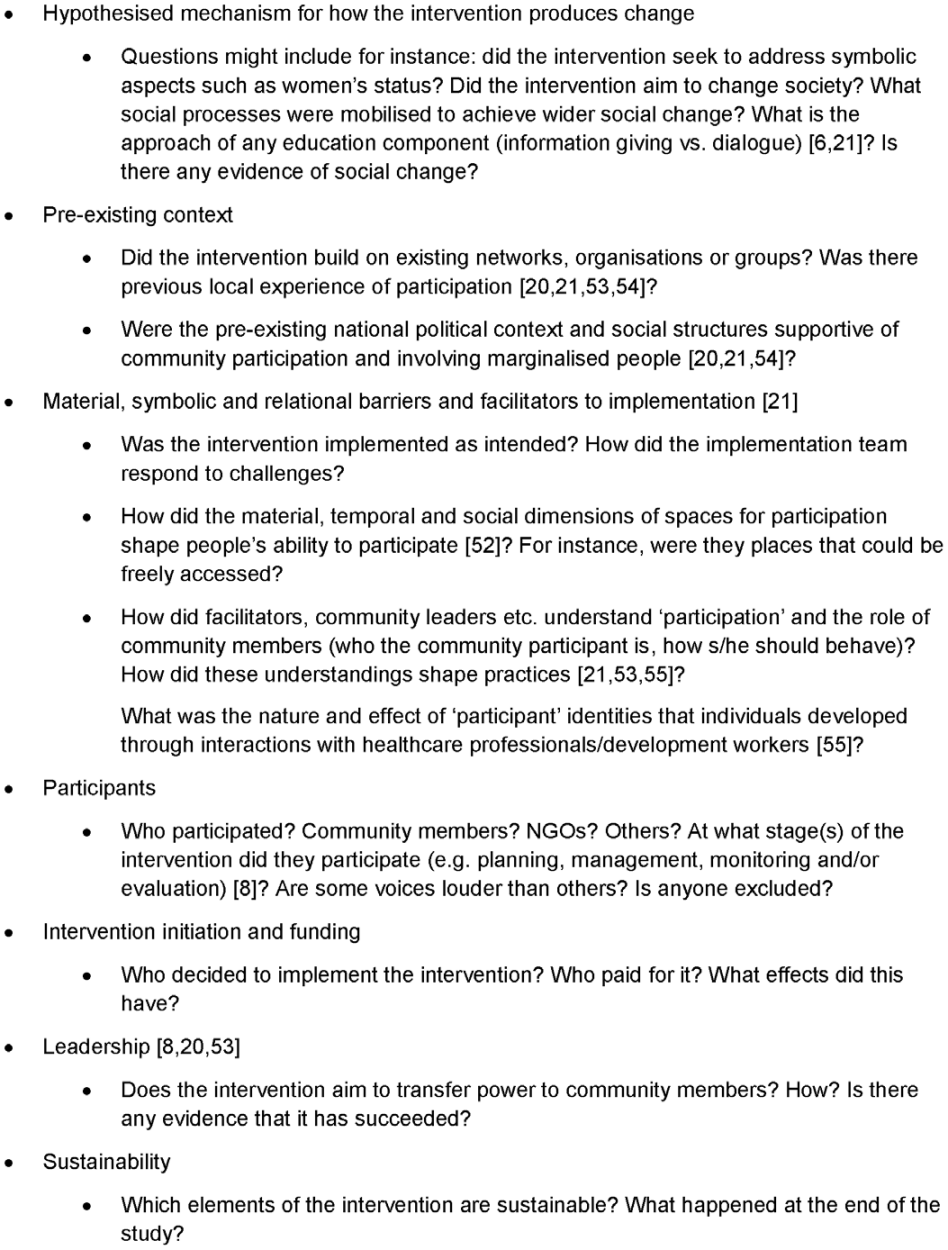
Key areas for qualitative investigation with examples of specific research questions.

We draw on previous work [6], [8], [20], [21], [52]–[55] to suggest key areas to consider in qualitative investigation of participation interventions (Figure 3). These include in-depth analysis of the mechanisms and processes through which the intervention might produce change [21], how contextual factors help or hinder these processes [21], and the nature and extent of the participation [8], [55]. Such qualitative work must be funded adequately to allow the careful analysis required and could increase overall costs. However, additional costs would be small compared with the cost of the RCT and the qualitative work should yield useful insights even if the trial delivers a negative result.

Lack of agreement on nomenclature within the literature meant that it was difficult to search precisely for articles on community participation. We tested our search terms against studies we were already aware of to ensure that they appeared, and found it hard to narrow our searches without missing relevant references. We may have missed items that were not indexed in databases. We presented an earlier version of this paper [56] and requested additional material from experts in attendance, but none was provided.

It is difficult to assess the likelihood of publication bias in our sample. However, it seems likely that small studies showing no effect of any given intervention may remain unpublished. The time, effort, and expense required to conduct full RCTs would probably encourage publication, regardless of outcome.

Most studies that examine the effects of community participation interventions on maternal and newborn health show benefits from participation. Unfortunately, the small number of high-quality studies, and a lack of information about why interventions have succeeded or failed, prevents us from stating what makes a participation intervention successful – an area of obvious interest for programme designers. Studies of participation interventions in other areas of health have the potential to illuminate this area further.

Future studies to assess the effects of community participation interventions – in maternal and newborn health, and more widely – should include both quantitative and qualitative approaches. The qualitative work should be at the heart of large trials of social interventions, and reported as a fundamental element of the findings. We have suggested key areas for qualitative data reporting alongside future quantitative studies of community participation interventions. Such qualitative investigation alongside RCTs will help us understand complex interventions in context, describe predicted and unforeseen impacts, assess potential for generalisability, and capture the less easily-measurable social and political effects of encouraging participation.

## Supporting Information

Table S1 **Appendix: details of the included studies.**
(PDF)

Checklist S1 **PRISMA flow diagram.**
(TIF)
