# Supplementary material for: Effects of Community Participation on Improving Uptake of Skilled Care for Maternal and Newborn Health: A Systematic Review
Source: PLoS One. 2013 Feb 4;8(2):e55012. doi: 10.1371/journal.pone.0055012 (PMC3563661; doi:10.1371/journal.pone.0055012)
Supplement: Table S1 — Appendix: details of the included studies. (PDF) [file pone.0055012.s001.pdf]

	Bangladesh: 3 districts, women's groups
Characteristics	Azad, K., S. Barnett, et al. (2010). "Effect of scaling up women's groups on birth outcomes in three rural districts in Bangladesh: a cluster-randomised controlled trial." Lancet 375(9721): 1193-1202.
Year (of intervention)	2005 - 2007
Study location	Three rural districts, Bangladesh
Brief description of intervention/study	Intervention clusters: facilitator convened women's groups to support participatory action and learning and develop and implement strategies to address maternal and neonatal health problems (intervention based on the work in Makwanpur, Nepal - see Manandhar et al). Of the nine intervention clusters, five received a traditional birth attendant intervention (providing basic training in how to conduct clean and safe deliveries, provision of safe delivery kits, recognition of maternal and infant danger signs, making emergency preparedness plans, mouth-to-mouth resuscitation, accompanying women to facilities), and four were controls for this. TBA intervention clusters also received additional training in bag-valve-mask resuscitation of neonates but control clusters only received the basic TBA training. 'Activities undertaken in both intervention and control clusters focused on improving referral systems, links between the community and health services and between different levels of health services, efficient use of available resources, basic and refresher clinical training relating to essential neonatal and maternal care, in addition to information, education, and communication materials' (p.1195).
Type/level of participation	Groups identified and prioritised maternal and neonatal problems to help to identify possible strategies, and to support planning, implementation, and monitoring of strategies in the community. Groups took part in a participatory learning and action cycle (p.1195).
Examines effects of community participation?	Yes
Pre-existing context	Other NGOs operating in the area offer financial incentives for individuals who work with them, thus potentially drawing female facilitators away (no financial incentives provided within the intervention). Pre-existing gender-based barriers in some of the intervention unions (e.g. lack of permission from husbands and in-laws) might have discouraged women from participating in groups, implementing strategies or seeking care.
Who initated the intervention?	Initiated externally by the authors.
Relational aspects (e.g. building community support, transferring leadership to participants etc.)	At the outset the research team approached community leaders and asked them for permission to implement the women's groups. They also conducted 451 community orientation meetings with members of union councils, community members, and community chairmen. Facilitators organised meetings and liaised with community leaders (with the support of supervisors). The authors report that problems retaining facilitators and supervisors 'might have led to disruptions in meetings and reduced support for community mobilisation' (p. 1200). Generally, coordinators lived away from facilitators which meant that facilitators did not receive refresher training and continuing support from them. The authors argue that the quality of the participatory intervention might have been affected by facilitators' lack of support from family decision-makers, especially their husbands.
Symbolic aspects (e.g. addressing women's status, types of social processes mobilised)	Women were encouraged to identify maternal and neonatal problems, and implement and evaluate strategies to resolve them. Strategies were different depending on the groups and also changed over time but the authors report that process assessment data showed that the main ones were: 'creation of emergency funds managed by the groups, the raising of awareness of maternal and newborn health issues during meetings and in the community by use of materials provided by the groups (such as picture cards and flipcharts), and the fostering of effective communication with health-care providers through meetings with group representatives.' (p.1199)

	Bangladesh: 3 districts, women’s groups
Characteristics	Azad, K., S. Barnett, et al. (2010). "Effect of scaling up women's groups on birth outcomes in three rural districts in Bangladesh: a cluster-randomised controlled trial." Lancet 375(9721): 1193-1202.
Material aspects (e.g. efforts to achieve sustained funding/symbolic support, enhancing access to material resources, seeking ways for participants to put their new skills into practice elsewhere)	No financial incentives were offered for participating in women's groups. The authors argue that this might have prevented some women from joining the groups in their local area where women regularly receive financial incentives to participate in NGO activities. Some women's groups were linked to NGOs. Women sought to address some of their problems through creating, implementing and managing emergency funds.
Leadership, planning and management	At the beginning, the women's groups only comprised women of reproductive age. However, later on, at the request of group members, mothers-in-laws, young people, and other women participated in the groups.
External support?	The study was funded externally by Women and Children First, the UK Big Lottery Fund, Saving Newborn Lives, and the UK Department for International Development.
Monitoring and evaluation done in participatory way?	Women were supported by facilitators in monitoring strategies.
Duration of intervention	The trial lasted three years.
Sustainability	Potential barriers to sustainability include the traditionally limited mobility of women and the lack of incentives (especially monetary) for female facilitators in the midst of other NGOs that give financial incentives.
Theoretical Framework	Not found in text.
Equity considered (PROGRESS)	Not found in text.
Cost considerations	Not found in text.
Study design	Low risk of bias. Cluster-randomised controlled trial. 18 clusters in total: 9 clusters randomly assigned (stratified randomisation) to intervention and 9 clusters randomly assigned to control condition. 5 intervention clusters also received TBA training intervention, the remaining 4 were controls for this. Within intervention clusters, outcomes were also compared between women who were women’s group members and those who were not.
Data collection	Data collected via prospective monitoring system similar to Ekjut trial (Tripathy et al., 2010; Barnett et al. 2008). All births and their outcomes recorded. TBAs identified all births and deaths in all women during pregnancy or up to 6 weeks after birth. Women then interviewed 6 weeks after birth to verify information found by TBAs. Also, questionnaire gathered data on women’s background characteristics, and the antenatal, delivery, and postpartum periods. Also, for stillbirths and neonatal deaths: verbal autopsy with mother; maternal deaths: verbal autopsy with relative/close friend.
Participants	Women aged 15 - 49 in the intervention clusters who resided in the study area and who had given birth during the study period.
Outcomes	Primary: NMR. Secondary: maternal deaths, stillbirths, uptake of antenatal and childbirth services, home-care practices during and after birth, infant morbidity, health-care seeking behaviour, perinatal mortality and early and late NMR.

	Bangladesh: 3 districts, women’s groups
Characteristics	Azad, K., S. Barnett, et al. (2010). "Effect of scaling up women's groups on birth outcomes in three rural districts in Bangladesh: a cluster-randomised controlled trial." Lancet 375(9721): 1193-1202.
Key Findings	No significant reduction in NMR in intervention clusters. Stillbirth rates did not differ between intervention and control clusters. No significant differences in most homecare practices or health-care-seeking behaviours. Proportion of institutional deliveries slightly higher in control than intervention clusters.

	Kenya
Characteristics	Kaseje, D., R. Olayo, et al. (2010). "Evidence-based dialogue with communities for district health systems' performance improvement." Glob Public Health 5(6): 595-610.
Year (of intervention)	2005 – 2007
Study location	Six districts of Nyanza Province (Kenya)
Brief description of intervention/study	Iterative dialogue and planning between service users and providers to improve service delivery, governance, management and health outcomes. Intervention included community participation component. Community Health Extension Workers (CHEWs) (professional) lived in community, facilitated community-level dialogue, supported CHWs (lay volunteers) and maintained Community Based Information System (CBIS). CHWs supported households in health improvement initiatives, maintained village register, facilitated household-level dialogue (see participation column). Community data e.g. on health displayed publically. Suggestion boxes with user satisfaction questionnaires established at health facilities.
Type/level of participation	Users and service providers participated in decision-making through committees at community, village and health facility level. Dialogue sessions held to discuss data collected through household registers and heath facilities, led by CHWs (monthly at household level) and by CHEWs (monthly at community level, and every 4 months at health facility levels, sub-district health stakeholders forums). Discussion of data followed by consensus building and action planning about unacceptable outcomes, needs improvement, actions to be implemented before the next dialogue session. Action planning was at household, community unit, health centre and sub-district levels.
Examines effects of community participation?	Yes. Tested an evidence-based dialogue model ('Assess, Dialogue, Plan, Act') for facilitating improvement of the District Health System (DHS) (in governance, management, service delivery) and the health of poor households that it served.
Pre-existing context	Not found in text.
Who initated the intervention?	The intervention was developed and tested by the authors.
Relational aspects (e.g. building community support, transferring leadership to participants etc.)	The authors report that the study was conducted in partnership with the Ministry of Health (MOH) and the communities. The University facilitated consultative workshops with the different stakholders (the provincial and district health management teams, community representatives and members of the local government administration). Research team members were directly involved in training and dialogue activities in partnership with DHS members.CHEWs functioned as facilitators of dialogue within the community, supported CHWs and maintained CBIS. CHWs were trained to support households in health improvement initiatives, to maintain the village register, and facilitate dialogue at the household level. The authors report that CHWs (supported by CHEWs) played a key role linking the household and the health system in the dialogue process and in 'bridging the gap' between demand and supply (p.609).The Kenyan MoH adopted the intervention model as a strategy for the implementation of the national Essential Package for Health. The implementation guidelines and training materials for rolling out the strategy were developed by the University.
Symbolic aspects (e.g. addressing women's status, types of social processes mobilised)	It is not clear from the paper but the authors report that the model was informed by theories of behaviour and that they wanted to test whether the intervention would lead to improvememts in self-efficacy (Bandura, 1997, 2000) which would facilitate change.The dialogue model 'was to provide a forum for exchange of information between the providers and clients.' (p. 608). Three-day training workshops were held three times during the intervention period. The aim of the workshops was to introduce the model to the health management teams, service providers and communities. The aim of the first workshop was to ensure the necessary skills to implement the intervention.

	Kenya
Characteristics	Kaseje, D., R. Olayo, et al. (2010). "Evidence-based dialogue with communities for district health systems' performance improvement." Glob Public Health 5(6): 595-610.
Material aspects (e.g. efforts to achieve sustained funding/symbolic support, enhancing access to material resources, seeking ways for participants to put their new skills into practice elsewhere)	Not found in text.
Leadership, planning and management	The iterative process of dialogue involved 'joint' assessment of available data, decision-making, planning and action by both service providers and communities. The study engaged the end users of services and communities in the design and implementation of research to improve the utilisation of results. Consensus on the conceptual framework and methodology of the study was developed through consultative workshops facilitated by the University and attended by different stakeholders (the provincial and district health management teams, community representatives and members of the local government administration). Committees were developed at the village, community and health facility level to bring together service users, providers and communities into decision-making. Community representatives participated in a dialogue process with managers and service providers on the data from health facilities, discussions towards consensus building regarding the data, prioritisation of elements for service improvement. They also participated in decision-making and planning on action towards improvement. CHWs facilitated household-level dialogue and supported households in health improvement initiatives. They also maintained village registers.
External support?	Prior to the intervention, consultative workshops were facilitated by the Great Lakes University of Kisumu, and attended by provincial and district health management teams, community representatives, and members of the local government administration. Funding provision not clear, although the whole intervention is a partnership between the University, the Kenyan Ministry of Health, and communities.
Monitoring and evaluation done in participatory way?	CHWs (lay volunteers) interpreted the household register data which was used for the dialogue process. The authors report that CHWs played a key role in 'collection and collation' (p. 609) of Community Based Information System (CBIS) which was used to prompt the dialogue process.
Duration of intervention	Two years. The length of dialogue sessions at household and community levels was an hour on average.
Sustainability	The study has led to the development of government policy that aims at strengthening the links between the health system and communities. The authors argue that this has created an enabling environment for rolling-out the model nationally as well as for continuing the process of implementing improvements.
Theoretical Framework	Theories of behaviour (e.g. Bandura,1997, 2000) and systems change (e.g. Oldham, 2004) informed the model. The authors wanted to test whether improvements in self-efficacy (Bandura, 1997, 2000) through the intervention would facilitate change.
Equity considered (PROGRESS)	Not found in text.
Cost considerations	Not found in text.

	Kenya
Characteristics	Kaseje, D., R. Olayo, et al. (2010). "Evidence-based dialogue with communities for district health systems' performance improvement." Glob Public Health 5(6): 595-610.
Study design	Moderate risk of bias. Participatory comparative, descriptive study. Service users, policy makers, provincial and district health management teams, community representatives, and members of the local government administration designed and implemented research via consultative workshops. Intervention sites (N=2 per district, total number of districts: 6). Control sites (N=2 per district) matched for geographical location and served by same health facility. NB intervention sites were selected for willingness to participate so bias likely (although there is no suggestion in the paper that the comparison areas were necessarily unwilling to participate)
Data collection	Surveys (household cluster sample structured questionnaires and health facility records) conducted in both intervention and control sites at the beginning of and two years after the intervention. Interviews with 2+ key informants per health facility (from governing and management structures). Focus group discussion with CHWs. Facility assessment tool used to assess governance, management and service delivery processes. Exit interviews with service users to measure satisfaction with 'services received during the study period, at baseline and follow-up' (p.601). Pre- and post- intervention assessments at start of 2005 and 2007 respectively.
Participants	The intervention study engaged service providers, the end users, policymakers and communities.
Outcomes	Measles immunisation (12-23 months), family planning (FP use), antenatal clinic attendance (4+) (Four or more ANC visits), health facility delivery, insecticide treated net (ITN) availability, ITN use, latrine presence, food availability, water treatment.
Key Findings	Improvement in intervention sites compared with control sites for: health facility childbirth (18% difference - control [23%] and intervention [41%], p= 0.000), measles immunisation (29% difference- control [58%] and intervention [87%] sites, p=0.000) (also: ITN use (27% difference - control [50%] vs. intervention [77%], p=0.000), latrine presence (21% difference - control [49%] vs. interventions [70%], p=0.0000), food availability (25% difference - control [39%] vs. intervention [64%], p=0.000), water treatment (16% difference - control [54%] vs. intervention [70%], p=0.000)). No significant differences in: ANC attendance, FP use, or ITN availability. Reports from intervention sites of building laboratories, maternity wings, toilets and water tanks, improved decision-making processes (p. 601).

Malawi (MaiMwana) women’s groups		
Characteristics	Rosato, M., Mwansambo, C., Lewycka, S., Kazembe, P., Phiri, T., Malamba, F., Newell, M., Osirin, D. & Costello, A. 2010. MaiMwana women’s groups: a community mobilisation intervention to improve mother and child health and reduce mortality in rural Malawi. Malawi Medical Journal, 22, 112-119.	Lewycka S. Reducing maternal and neonatal deaths in rural Malawi: Evaluating the impact of a community based women’s group intervention [Thesis]: University College London; 2010.
Year (of intervention)	2005 - 2010	See Rosato et al.
Study location	Mangochi district, Malawi	See Rosato et al.
Brief description of intervention/study	Aim is to build the capacities of communities to take control of mother and child health issues that affect them. In the first arm of the study trained local female facilitators established women's groups and used participatory rural appraisal tools to guide participants through a community action cycle to identify and implement solutions to MNCH problems (p.112). A second arm contained an intervention involving volunteer infant feeding and care counsellors to promote behaviour change. 48 clusters were divided equally into four arms: 1. Women's groups only 2. Counselling only 3. Both women's groups and counselling 4. None-control.	See Rosato et al. Emphasis is on evaluating the impact of the women's group arm only.
Type/level of participation	High level of community participation. Despite being conceptualized externally, within the intervention, women's group members are responsible for identifying local problems with MNCH, designing strategies to address them, mobilising local resources and/or capacities and/or capitalising on partnerships to implement strategies, and then evaluating the strategies.	See Rosato et al.
Examines effects of community participation?	Yes, but results not reported in this paper.	Yes
Pre-existing context	Not found in text.	Prior to the intervention, three levels of community sensitisation took place with key stakeholders, introducing them to the aims of the MaiMwana project. The Mchinji District Executive Committee, Mchinji District Assembly, Area Development Committees, and Village Development Committees were all involved in community entry activities.
Who initated the intervention?	Externally initiated by the authors.	See Rosato et al.

Malawi (MaiMwana) women’s groups		
Characteristics	Rosato, M., Mwansambo, C., Lewycka, S., Kazembe, P., Phiri, T., Malamba, F., Newell, M., Osirin, D. & Costello, A. 2010. MaiMwana women’s groups: a community mobilisation intervention to improve mother and child health and reduce mortality in rural Malawi. Malawi Medical Journal, 22, 112-119.	Lewycka S. Reducing maternal and neonatal deaths in rural Malawi: Evaluating the impact of a community based women’s group intervention [Thesis]: University College London; 2010.
Relational aspects (e.g. building community support, transferring leadership to participants etc.)	Intervention has a strong advocacy component, whereby groups are linked to appropriate organisations, locally, regionally, or nationally (p.115). Intervention encourages the engagement of both women and men, although with the inclusion of women being more prominent.	Men were encouraged to participate in the women’s groups, sometimes taking on prominent roles like being the secretary or chairperson. The inclusion of men may have influenced the way women participated, although men are described to have been particularly useful in implementing chosen strategies. When men were present there was an observed tendency for women to talk less and adopt a more 'traditional deferential role' (p.231) in relation to men.
Symbolic aspects (e.g. Addressing women's status, types of social processes mobilised)	Zonal facilitators (who are recruited locally from within their cluster) are trained extensively in community mobilisation techniques, advocacy, and the liaising of groups that can offer appropriate support for strategies. Such skills are transferrable across many issues within and beyond health.	See Rosato et al.
Material aspects (e.g. Efforts to achieve sustained funding/symbolic support, enhancing access to material resources, seeking ways for participants to put their new skills into practice elsewhere)	By nature of the intervention, the strategies designed in community mobilisation action cycles to address MNCH were those that could feasibly address the priority problems identified by making best use of local resources (p.114). The project does not provide additional resources to implement strategies; groups must draw on community mobilisation, local capacities, or relationships with government and non-governmental organizations for support (p.115).	See Rosato et al.
Leadership, planning and management	Local MNCH strategies are entirely designed, implemented, and evaluated by community members. The women's groups are facilitated (not led) by zonal facilitators, who are recruited and salaried women selected for their membership within one of the villages within their cluster and experience in education. Recruited and salaried supervisors monitor the work of zonal facilitators.	See Rosato et al.
External support?	The intervention was initially funded by Saving Newborn Lives and a Wellcome Trust Strategic Award and later by DFID and UNICEF Malawi.	See Rosato et al.

Malawi (MaiMwana) women’s groups		
Characteristics	Rosato, M., Mwansambo, C., Lewycka, S., Kazembe, P., Phiri, T., Malamba, F., Newell, M., Osirin, D. & Costello, A. 2010. MaiMwana women’s groups: a community mobilisation intervention to improve mother and child health and reduce mortality in rural Malawi. Malawi Medical Journal, 22, 112-119.	Lewycka S. Reducing maternal and neonatal deaths in rural Malawi: Evaluating the impact of a community based women’s group intervention [Thesis]: University College London; 2010.
Monitoring and evaluation done in participatory way?	M&E of the intervention itself was done by the authors. However, the community-level strategies that will ultimately drive the desired outcomes are evaluated in a participatory manner as part of phase four of the intervention, 'Evaluating Together', in which the group evaluates their strategies and plans for the future (p.114).	See Rosato et al.
Duration of intervention	2005 - 2010	See Rosato et al.
Sustainability	Reliance on local knowledge, resources, and connections ensures that the intervention can, theoretically, continue beyond external support.	See Rosato et al.
Theoretical Framework	Not found in text.	Not found in text.
Equity considered (PROGRESS)	Not found in text.	Not found in text.
Cost considerations	Major resource inputs were for salaries of zonal facilitators (24 facilitators, £40/month), and supervisors (five total, £200 - £350/month) plus training expenses, equipment, office space, and other materials to support the program (p.115).	See Rosato et al.
Study design	Low risk of bias. Cluster-randomised controlled trial with 48 clusters evenly assigned to four arms (p.113): 1. Women's groups only 2. Counselling only 3. Both women's groups and counselling 4. None-control.	See Rosato et al. This thesis was an impact evaluation of the women's groups on the outcomes indicated below.
Data collection	Baseline data collected in 2004 from a population of 145,426 (a purposive sub-sample from the total population of approximately 480,000). Demographic, MNCH care, and MNCH care-seeking data collected from women who had become pregnant from January - June 2005 (n=1399) (p.118/119).	Monthly pregnancy surveillance to detect new pregnancies; birth notification; one-month post-partum interviews; maternal and/or stillborn/neonatal autopsy (if mother or baby died); six-month post-partum interviews; re-census of households in study areas (done in May/June 2008); and infant follow-ups for all babies born after January 1st, 2005.
Participants	Primary participants are women of childbearing age (15 - 49), secondary participants are women younger than 15 or older than 49, and tertiary participants are men and boys (p.113).	See Rosato et al.

Malawi (MaiMwana) women’s groups		
Characteristics	Rosato, M., Mwansambo, C., Lewycka, S., Kazembe, P., Phiri, T., Malamba, F., Newell, M., Osirin, D. & Costello, A. 2010. MaiMwana women’s groups: a community mobilisation intervention to improve mother and child health and reduce mortality in rural Malawi. Malawi Medical Journal, 22, 112-119.	Lewycka S. Reducing maternal and neonatal deaths in rural Malawi: Evaluating the impact of a community based women’s group intervention [Thesis]: University College London; 2010.
Outcomes	Overall outcomes of the intervention described: reduced maternal, neonatal, and infant morbidity and mortality.	Primary: maternal, perinatal, neonatal, and infant mortality. Secondary: Maternal and infant morbidity, changes in caretaker practices (hygiene, use of ITNs, early and exclusive breastfeeding, etc.), changes in care-seeking behaviour (antenatal care, uptake of PMTCT, postnatal care, etc.)
Key Findings	Of 207 established women's groups, 197 are still active, 8 chose to disband, and 2 joined nearby groups. Over 12,000 men and women attended the groups at least once, with 8,000, 6,000, 1, 000, and 7,00 attending phases 1, 2, 3, and 4 respectively (men invited to 3 and 4 only).	The findings of the study are principally negative, i.e. the intervention does not have a measured effect on the outcomes of interest. There was no decline in maternal or neonatal mortality associated with the intervention. Odds of having any antenatal care in health facility were higher in the intervention group but of marginal statistical significance(Adj OR 1.44, 95%CI 1.00-2.13). Odds were lower of having a TBA attended birth (Adj OR 0.71, 95%0.51-0.97) but as there is no corresponding increase in births with a skilled provider, it is hard to assess what this might mean in terms of improved maternal safety. (All statistics from Table 5.13, p.181) When the four arms were analysed separately, there was some evidence that the women's group only arm was associated with a reduction in perinatal mortality in years 2-3 (Adj OR 0.64 (95%CI 0.44-0.94) compared with the control arm) (Table 5.15, p.187) Again, while positive, this is difficult to interpret because there is no effect for all three years combined, and when analysed together with the arm containing women's group and infant feeding combined, there is no significant effect.

	Nepal young people	
Characteristics	Malhotra A, Sanyukta M, Rohini P, Roca E. Nepal: the distributional impact of participatory approaches on reproductive health for disadvantaged youths. Washington, DC: World Bank (Human Development Network) Health, Nutrition, and Population, 2005.	Mathur, S., R. Pande, et al. (2005). Community mobilization and the reproductive health needs of married adolescents in South Asia, [Unpublished] 2005. Presented at the 2005 Annual Meeting of the Population Association of America, Philadelphia, Pennsylvania, March 31 - April 2, 2005: 32.
Year (of intervention)	2000-2003	1998 - 2003
Study location	Nepal	Kathmandu and Nawalparasi district, Nepal
Brief description of intervention/study	Intervention sites: community-based, client-centred, participatory approach aimed at improving sexual and reproductive health of young people ('adolescents') using participatory methodologies and techniques (e.g. committees, task forces and youth clubs) for research, needs assessment, intervention design, implementation, monitoring, and evaluation phases. Participatory activities that were implemented: community mapping, mobility mapping, free listing and ranking, lifelines, body mapping, reproductive health problem trees, and reproductive health service matrix construction. Control sites: more traditional (i.e. non-participatory) reproductive health research, design, and intervention.	See Malholtra et al.
Type/level of participation	Participation happened at different stages of the intervention from the design and needs assessment to the implementation, monitoring and evaluation. Participatory mechanisms and structures (e.g. advisory and coordination teams and consultative committees) were established to build 'community ownership and involvement' at every stage of the intervention (p.214)	See Malholtra et al.
Examines effects of community participation?	Assesses the impact of a mix of participatory methodologies and techniques in improving youth reproductive health. The intervention was linked to other programmes, which had been developed and prioritised in a participatory way too.	Yes, particularly with regard to its effect on changing social norms surrounding reproductive health and the attitudes of healthcare providers towards young people interested in reproductive health services.
Pre-existing context	Not found in text.	In the study area, women are often made to stay at home and so are unable to access information and health services. Major life and health decisions for women are typically made by husbands and mothers-in-law.

	Nepal young people	
Characteristics	Malhotra A, Sanyukta M, Rohini P, Roca E. Nepal: the distributional impact of participatory approaches on reproductive health for disadvantaged youths. Washington, DC: World Bank (Human Development Network) Health, Nutrition, and Population, 2005.	Mathur, S., R. Pande, et al. (2005). Community mobilization and the reproductive health needs of married adolescents in South Asia, [Unpublished] 2005. Presented at the 2005 Annual Meeting of the Population Association of America, Philadelphia, Pennsylvania, March 31 - April 2, 2005: 32.
Who initated the intervention?	Externally initiated. The authors report that the project was a collaboration between two international organisations (service delivery organisation 'EngenderHealth', research organisation 'International Center for Research on Women'), and local Nepali NGOs ('New ERA Ltd.' and 'BP Memorial Health Foundation').	See Malholtra, et al.
Relational aspects (*e.g.* building community support, transferring leadership to participants etc.)	The project was a collaboration of local Nepali NGOs and two international organisations (research and service delivery organisations). The intervention in the study site sought to address various barriers to reproductive health (structural, normative, and systemic) linking youth reproductive health programmes with others, and shape the everyday environments of young people (adult education, activities to address social norms, economic livelihood interventions, etc.). The control sites only focused on addressing immediate risk factors such as sexually transmitted infections and unwanted pregnancies. The authors report that in the intervention site social networks for the exchange of information and counseling were strengthened (p. 230). The intervention actively sought to build decision-making structures and coalitions as well as imparting information. The participatory structures of the study site built community skills in: decision-making; consensus building; planning; organising; consulting; negotiating (e.g. with the village development committee); and demanding accountability and resources from various actors (e.g. government funds to continue project activities).	Within the intervention, efforts were made to design strategies to link 'youth reproductive health programs with other programs that were deemed to influence the environment youth lived in' (p.9). Community infrastructure incorporated to coordinate the project, e.g. adolescent coordination teams and parent advisory committees. Youth and adult community members worked together to identify and prioritize interventions. In intervention sites, local service providers were trained, including traditional healers and family and child health volunteers, to address issues of lack of mobility that young women may face when trying to address services outside of their community.

	Nepal young people	
Characteristics	Malhotra A, Sanyukta M, Rohini P, Roca E. Nepal: the distributional impact of participatory approaches on reproductive health for disadvantaged youths. Washington, DC: World Bank (Human Development Network) Health, Nutrition, and Population, 2005.	Mathur, S., R. Pande, et al. (2005). Community mobilization and the reproductive health needs of married adolescents in South Asia, [Unpublished] 2005. Presented at the 2005 Annual Meeting of the Population Association of America, Philadelphia, Pennsylvania, March 31 - April 2, 2005: 32.
Symbolic aspects (*e.g.* addressing women's status, types of social processes mobilised)	In the intervention site there was a focus on 'changing fundamental norms and institutions', which was a key factor in increasing disadvantaged people's demand for services and information (p.231). The authors also report that the participatory approach of the intervention led to 'a new mindset in the communities marked by a deeper, more sophisticated understanding of youth reproductive health and its implications' (p.232) (e.g. an understanding of how family, gender, and social structures and norms limit healthier sexual and reproductive behaviours). Community involvement built via advisory and coordination teams and consultative committees to engage community members (young people and older adults) with an emphasis on disadvantaged groups. Project staff used strategies such as 'rotating representation' to ensure the active participation of 'disempowered groups' (e.g. the poor, women and ethnic minorities) (p. 215). Community task forces created to establish priorities and design interventions.	Behaviour and attitudes of older adults were targeted within the intervention, because of their influential role for young people. Strategies were designed to link young people to 'adult education programs, activities to address social norms, and economic [livelihood] interventions' (p.9). Within the intervention, 'reproductive health outcomes were conceptualized to exist within a context of young people's ideals and aspirations and the broader social institutions and norms that define, shape and constrain life outcomes and choices for young women and men' (p.7). Authors report that through involvement in community-level committees, young people and older adults were able to participate in project decision making. The intervention 'enabled young women to gain confidence and tools to better articulate their own reproductive health needs, positively influenced attitudes of key gate-keepers such as husbands and in-laws about the need for young pregnant women to seek care, and changed provider attitudes and approachability for youth' (p.21). In order to challenge pre-exisiting social beliefs, men were targeted through separate mobile seminars on sexual and reproductive health.
Material aspects (*e.g* . efforts to achieve sustained funding/symbolic support, enhancing access to material resources, seeking ways for participants to put their new skills into practice elsewhere)	Not found in text.	Not found in text.

	Nepal young people	
Characteristics	Malhotra A, Sanyukta M, Rohini P, Roca E. Nepal: the distributional impact of participatory approaches on reproductive health for disadvantaged youths. Washington, DC: World Bank (Human Development Network) Health, Nutrition, and Population, 2005.	Mathur, S., R. Pande, et al. (2005). Community mobilization and the reproductive health needs of married adolescents in South Asia, [Unpublished] 2005. Presented at the 2005 Annual Meeting of the Population Association of America, Philadelphia, Pennsylvania, March 31 - April 2, 2005: 32.
Leadership, planning and management	The authors report that the design and implementation of the intervention in the study sites were more 'inclusive, and interactive, with a great deal of attention to building community ownership and involvement at every step.' (p.214). Action planning conducted at the intervention design stage was participatory. The results of the needs assessments exercise were 'shared and analyzed with the community, and community task forces were created to set priorities and design feasible interventions' (p.215). In the study sites, adults and young people were allowed to increase their 'authority and decision-making power in the project' through community-level committees (p. 215). The other programmes linked to intervention in the study sites were developed and prioritized by community members.	Youth participation was a key strategy for community mobilization [the study] engaged young people in needs assessment, program design, implementation, and assessment' (p.6). Local structures (including NGOs and community member-led organizations, etc.) were used to implement programme activities.
External support?	Not reported in text.	External NGOs (EngenderHealth), academic institutions (the International Centre for Research on Women), and local NGOs (New ERA and BP Memorial) collaborated to implement the project. Funding sources not reported.
Monitoring and evaluation done in participatory way?	The authors report that 'participatory techniques' were employed in monitoring and evaluation through 'advisory and coordination teams and consultative committees that engaged youth and adult community members, especially those who were disadvantaged.' (p.214-215)	Young people were involved in assessment of the programmes as part of the intervention design. The overall evaluation of the programme's effectiveness in achieving outcomes was conducted by the authors.
Duration of intervention	From 12 to 24 months (intervention rolled out in sets with the first beginning in November 2000 and the last ending in March 2003).	See Malholtra et al.
Sustainability	The authors report that the intervention led to enhanced understanding of how different factors (family, gender, social structures and norms) constrain healthier reproductive and sexual heath, which they argue is an indication of the sustainability of the demand for youth reproductive health services.	The reliance on locally-established organizations allowed for 'ownership and sustainability' (p.29). However, a threat to sustainability is that the existing health infrastructure will likely not be able to keep up with increased demand for youth reproductive health services (p.29).

	Nepal young people	
Characteristics	Malhotra A, Sanyukta M, Rohini P, Roca E. Nepal: the distributional impact of participatory approaches on reproductive health for disadvantaged youths. Washington, DC: World Bank (Human Development Network) Health, Nutrition, and Population, 2005.	Mathur, S., R. Pande, et al. (2005). Community mobilization and the reproductive health needs of married adolescents in South Asia, [Unpublished] 2005. Presented at the 2005 Annual Meeting of the Population Association of America, Philadelphia, Pennsylvania, March 31 - April 2, 2005: 32.
Theoretical Framework	Authors claim that the study was aimed at examining whether participatory interventions can lead to increased empowerment of and accountability to poor and disadvantaged people, and improve health outcomes and service accessibility for this population. They also argue that participatory interventions are more likely to have a positive impact on young people's critical thinking and decision-making capacities (p.213).	Not reported in text.
Equity considered (PROGRESS)	Study targeted youth reproductive health because young people disadvantaged when tying to access reproductive services and information, particularly in Nepal. Authors report that the rural-urban difference in the selection of the intervention and control sites was aimed at taking into account structural and wealth differentials and claim that the intervention reduced inequities.	Not reported in text.
Cost considerations	Not reported in text.	Not reported in text.
Study design	Moderate risk of bias. Intervention in two study sites (one urban and one rural) compared with two control sites (urban and rural). 'Communities selected were randomly assigned to study or control' (p.214). Study site interventions aimed to address structural, normative, and systemic barriers to youth reproductive health and were developed and prioritized by community members. Control sites focused only on the most immediate risk factors (e.g. sexually transmitted infections and unwanted pregnancies). Control sites did not employ participatory or collaborative methods (were developed and implemented by project staff) and received reproductive health interventions through adolescent-friendly services, peer education and counselling, and teacher education.	See Malholtra et al.

	Nepal young people	
Characteristics	Malhotra A, Sanyukta M, Rohini P, Roca E. Nepal: the distributional impact of participatory approaches on reproductive health for disadvantaged youths. Washington, DC: World Bank (Human Development Network) Health, Nutrition, and Population, 2005.	Mathur, S., R. Pande, et al. (2005). Community mobilization and the reproductive health needs of married adolescents in South Asia, [Unpublished] 2005. Presented at the 2005 Annual Meeting of the Population Association of America, Philadelphia, Pennsylvania, March 31 - April 2, 2005: 32.
Data collection	Data collected through household survey at baseline and endline and analysed as a cross-sectional sample, not a longitudinal sample, as participants were not followed throughout the study. Qualitative data also collected. For prenatal care and institutional childbirth information, young unmarried women of the study areas (14-21-year-olds at baseline and 18-25-year-olds at endline) were compared. Also see Mathur et al.	Quantitative data were collected through household baseline and endline surveys conducted in 1999 and 2003 respectively. At baseline, 373 young people were interviewed and at endline, 359 were interviewed, of whom 84 and 81, respectively, were married. Qualitative data were also collected from young married and unmarried men and women, adults, and health service providers through focus group discussions, in-depth interviews, key informant interviews, community mapping, making lifelines, body mapping, creating reproductive health problem trees, and reproductive health service matrices. Also see Malholtra et al.
Participants	14-21-year-olds at baseline, 14-25-year-olds at endline, living in the study sites: two rural sites in Nawalparasi district and Kawasoti district and two urban sites in middle-class suburbs of Kathmandu.	Targets of the intervention were youth aged 14 - 21, but participants within programming included young married women, their partners, older men and women, and health service providers.
Outcomes	Prenatal care in first pregnancy of unmarried women (whether she visited a trained provider, i.e. doctor, nurse, trained clinician, for prenatal care in her first pregnancy at least once); and institutional childbirth (whether the birth, miscarriage or abortion resulting from first pregnancy was at a medical facility). Other outcome measures not relevant to this review also reported.	Primary: Maternal care, specifically knowledge and use of antenatal, childbirth, and post-natal care by young married women. Secondary: improved reproductive health knowledge among male partners, improved attitudes and care from health care providers regarding reproductive health for youth.

	Nepal young people	
Characteristics	Malhotra A, Sanyukta M, Rohini P, Roca E. Nepal: the distributional impact of participatory approaches on reproductive health for disadvantaged youths. Washington, DC: World Bank (Human Development Network) Health, Nutrition, and Population, 2005.	Mathur, S., R. Pande, et al. (2005). Community mobilization and the reproductive health needs of married adolescents in South Asia, [Unpublished] 2005. Presented at the 2005 Annual Meeting of the Population Association of America, Philadelphia, Pennsylvania, March 31 - April 2, 2005: 32.
Key Findings	Pregnant women receiving prenatal care for their first pregnancy declined in study site from baseline to endline (71.4% to 58.8%), and increased slightly in control site (53.6% to 56.8%). Small increases in women in the study site giving birth in institutions between baseline and endline (48.2% to 51.9%), but greater increase in control group (32.1% to 47.4%). Statistical tests for significance of these differences not presented. Authors claim intervention successful in reducing advantage-based differentials in youth reproductive health outcomes (e.g. urban-rural differences in pregnant women accessing prenatal care declined in the intervention groups pre- and post- study from OR: 16.4 (p=0.001) to 1.2 (p=0.644). In the control group the decline was much smaller, from OR: 3.7 (p=0.028) to 3.2 (p=0.021). Similarly, for institutional births, the urban-rural difference declined in the intervention group from OR: 15.6 to 4.6 (p=0.000, 0.002) but increased in control group OR: 13.5 to 21.3 (p=0.000)). The authors draw on qualitative data to explain some of the factors that shaped the success of the participatory approach: '(1) facilitating the co-production of services; (2) empowering youths and adults and increasing the accountability of service providers and policy makers to the communit; and (3) increasing community demand for information and services.' (p.230)	Overall, the participatory intervention was 'only marginally more effective in changing women’s knowledge and awareness of maternal care issues, but was more effective in increasing awareness about where maternal care services could be found, and changing practices around use of services' (p.12). Some raw data are provided on outcome measures (e.g. Tables 3-5), but no statistical analysis is presented.

Nepal: Makwanpur women’s groups			
Characteristics	Manandhar, D. S., D. Osrin, et al. (2004). "Effect of a participatory intervention with women' groups on birth outcomes in Nepal: cluster-randomised controlled trial." Lancet 364(9438): 970-979.	Morrison J, Tamang S, Mesko N, Osrin D, Shrestha B, Manandhar M, et al. Women's health groups to improve perinatal care in rural Nepal. BMC Pregnancy Childbirth. 2005;5(6):1-12.	Wade A, Osrin D, Shrestha BP, Sen A, Morrison J, Tumbahangphe KM, et al. Behaviour change in perinatal care practices among rural women exposed to a women's group intervention in Nepal. BMC Pregnancy Childbirth. 2006;6(20):1-10.
Year (of intervention)	2001 - 2003	2001 - 2003	2001 - 2003
Study location	Makwanpur district, Nepal	Makwanpur district, Nepal	Makwanpur district, Nepal
Brief description of intervention/study	Intervention arm: Facilitated women's groups first discussed issues around childbirth and care behaviours in the community and then developed, implemented, and assessed strategies, which included community-generated funds for maternal or infant care, stretcher schemes, production and distribution of clean childbirth kits, home visits by group members to newly pregnant mothers, and awareness-raising with a locally made film to create a forum for discussion. Also health-service strengthening activities. Control arm: health-service strengthening activities alone.	Employed trained local female facilitators to organise, generate interest in, and facilitate women's group meetings in each intervention site. Supervisors (nationally advertised) were also employed (1 supervisor to support 3 facilitators). 111 women's groups were established. Initial meetings focused on 'problem identification' through facilitated discussion of the reasons (social and medical) for neonatal and maternal deaths in the community, leading to groups identifying three priority problems of neonates and/or pregnancy. Following problem prioritisation, groups progressed to 'planning together' strategies for responding to these problems, and later, through a community meeting, the wider community was invited to this planning process.	See Manandhar et al. 2004
Type/level of participation	Women's groups facilitated by a literate local woman who received training in perinatal health issues and participatory communication techniques. Groups convened once a month. Only 8% of all respondents participated in the women's groups, but 37% of newly pregnant women participated. Women's group members, supported by the trained facilitator, developed, implemented, and assessed strategies.	Local women attending regular facilitated and supervised group meetings and were involved in identifying problems, developing and implementing strategies to respond to these problems, and in participatory learning.	See Manandhar et al. 2004 and Morrison et al. 2005.
Examines effects of community participation?	Yes	Describes the intervention process.	Yes

Nepal: Makwanpur women's groups			
Characteristics	Manandhar, D. S., D. Osrin, et al. (2004). "Effect of a participatory intervention with women' groups on birth outcomes in Nepal: cluster-randomised controlled trial." Lancet 364(9438): 970-979.	Morrison J, Tamang S, Mesko N, Osrin D, Shrestha B, Manandhar M, et al. Women's health groups to improve perinatal care in rural Nepal. BMC Pregnancy Childbirth. 2005;5(6):1-12.	Wade A, Osrin D, Shrestha BP, Sen A, Morrison J, Tumbahangphe KM, et al. Behaviour change in perinatal care practices among rural women exposed to a women's group intervention in Nepal. BMC Pregnancy Childbirth. 2006;6(20):1-10.
Pre-existing context	Some facilitators worked with existing women's groups, but there is no information on previous community participation activities within the community or these groups.	Female community health volunteers develop health promotion activities through women's groups. Some of these existing groups were used for the intervention (Manandhar, et al., 2004).	Not found in text. See Manandhar et al. 2004.
Who initated the intervention?	Agents external to the community (authors).	Unclear from text, but authors say 'we postulated that a community-based participatory intervention could reduce the neonatal mortality rate' (p.971) implying that they (or some of them) initiated the intervention.	Not found in text. See Manandhar et al. 2004 and Morrison et al. 2005.

Nepal: Makwanpur women's groups			
Characteristics	Manandhar, D. S., D. Osrin, et al. (2004). "Effect of a participatory intervention with women' groups on birth outcomes in Nepal: cluster-randomised controlled trial." Lancet 364(9438): 970-979.	Morrison J, Tamang S, Mesko N, Osrin D, Shrestha B, Manandhar M, et al. Women's health groups to improve perinatal care in rural Nepal. BMC Pregnancy Childbirth. 2005;5(6):1-12.	Wade A, Osrin D, Shrestha BP, Sen A, Morrison J, Tumbahangphe KM, et al. Behaviour change in perinatal care practices among rural women exposed to a women's group intervention in Nepal. BMC Pregnancy Childbirth. 2006;6(20):1-10.
Relational aspects (e.g. building community support, transferring leadership to participants etc.)	Before the intervention, the authors held meetings with the Makwanpur District Development Committee, the Chief District Officer, and local stakeholders. The study clusters were village development committees. One of the reasons given: representatives functioned as key points of liaison. Community leaders helped identify potential female facilitators. Word of mouth within the community was also used. Support for the intervention from other community members was planned together and sought by the women's groups. Partnership with the District Public Health Office to train female community health volunteers, TBAs, and government health staff in newborn care. There were security problems (third year of study), which meant that some women's groups had to postpone meetings. Women's group strategies involved interaction outside the group, which the authors say increased awareness of perinatal health-related issues.	Linkages established with community leaders, non-governmental organisations, and district health services. Authors report that 'close' alliances with community leaders and community health workers helped implementation (p.1). Before implementation, the authors sought but did not find local NGOs or community based organisations that 'routinely' worked in all the study areas; these organisations had 'different agendas' (p.3). Community support ('awareness and interest') for the intervention was sought by group facilitators and supervisors (p.3). Support from the community sought through community meetings planned and organised by women's groups to 'legitimize' their work (p.5), and community (including leaders) involved in planning strategies. Lack of continuity for some groups was reportedly: lack of support from local leaders, husbands, and health workers, and an unstable security environment. When local health personel and chairmen participated, community meetings to plan strategies together, discussions 'were livelier and planning more productive' (p.6). Local female community health volunteers (lowest cadre of government-appointed health staff) and TBAs were actively involved in the women's groups, allowing the volunteers to have contact with a user group and conduct their health education work. See also Manandhar et al. (2004).	Not found in text. See Manandhar et al. 2004 and Morrison et al. 2005.

Nepal: Makwanpur women’s groups			
Characteristics	Manandhar, D. S., D. Osrin, et al. (2004). "Effect of a participatory intervention with women' groups on birth outcomes in Nepal: cluster-randomised controlled trial." Lancet 364(9438): 970-979.	Morrison J, Tamang S, Mesko N, Osrin D, Shrestha B, Manandhar M, et al. Women's health groups to improve perinatal care in rural Nepal. BMC Pregnancy Childbirth. 2005;5(6):1-12.	Wade A, Osrin D, Shrestha BP, Sen A, Morrison J, Tumbahangphe KM, et al. Behaviour change in perinatal care practices among rural women exposed to a women's group intervention in Nepal. BMC Pregnancy Childbirth. 2006;6(20):1-10.
Symbolic aspects (e.g. addressing women's status, types of social processes mobilised)	Use of action-learning cycles in women's groups to: prompt social dialogue about local knowledge and beliefs; identify and prioritise problems; and encourage women to formulate strategies. The intervention was not about ‘teaching’ and transferring health-related information. Authors emphasise the idea that the facilitator should act as a catalyzing agent for change and ‘broker of information’ rather than as a teacher or instructor. Women attending the group had an active role.	The intervention sought a social process of ‘learning together’ about perinatal problems, which involved dialogue within the groups and with other community members outside the groups (women were encouraged to talk with neighbours and friends).	Not found in text. See Manandhar et al. 2004 and Morrison et al. 2005.
Material aspects (e.g. efforts to achieve sustained funding/symbolic support, enhancing access to material resources, seeking ways for participants to put their new skills into practice elsewhere)	Women were not given money to participate in the women's group activities (sustainability reasons given by sponsors). Women developed strategies which capitalised on local resources (e.g. The development of a local emergency fund, stretcher schemes, production of clean childbirth kits, etc.).	Women’s group female facilitators were paid a salary ‘slightly higher than the government equivalent’ (p.3). Women were encouraged to find strategies (to identified problems) which would use local resources. 23 months after the first health fund, women's groups had generated between 10.5 and 133.8 US dollars (731-9635 rupees). The profits made from the locally produced home childbirth kits went to the mother and child health fund.	See Manandhar et al. 2004 and Morrison et al. 2005.

Nepal: Makwanpur women’s groups			
Characteristics	Manandhar, D. S., D. Osrin, et al. (2004). "Effect of a participatory intervention with women' groups on birth outcomes in Nepal: cluster-randomised controlled trial." Lancet 364(9438): 970-979.	Morrison J, Tamang S, Mesko N, Osrin D, Shrestha B, Manandhar M, et al. Women's health groups to improve perinatal care in rural Nepal. BMC Pregnancy Childbirth. 2005;5(6):1-12.	Wade A, Osrin D, Shrestha BP, Sen A, Morrison J, Tumbahangphe KM, et al. Behaviour change in perinatal care practices among rural women exposed to a women's group intervention in Nepal. BMC Pregnancy Childbirth. 2006;6(20):1-10.
Leadership, planning and management	The intervention was introduced by agents external to the community, but target women identified, prioritised and implemented strategies.	Trial intervention introduced by external agents (MIRA). Group facilitators selected through interviews by MIRA employees. Supervisors were also recruited through formal interviews but not clear from the text whether MIRA employees were also involved. Although female facilitators were trained to use a meeting facilitation manual adapted from the Warmi project in Bolivia, they were also 'allowed scope for their own input' (p.3). Both women’s groups and community members participated in the planning together stage of finding strategies for identified problems. The authors report that 'it is highly likely that the facilitation team's attempts to adequately support the facilitators may have led to less participatory processes taking place, especially in the case of strategy development.' (p.9). The manual was developed as a ‘reference guide’ but was regarded by the facilitation team as 'an essential resource' and 'an instruction booklet'.	See Manandhar et al. 2004 and Morrison et al. 2005.
External support?	Study funded by agents external to the community.	Study funded by agents external to the community: the British Government Department for International Development, UNICEF Nepal, and theWorld Health Organisation's Division of Child and Adolescent Health, with additional financial assistance from UNICEF Nepal and UNFPA Nepal.	See Manandhar et al. 2004 and Morrison et al. 2005.

Nepal: Makwanpur women's groups			
Characteristics	Manandhar, D. S., D. Osrin, et al. (2004). "Effect of a participatory intervention with women' groups on birth outcomes in Nepal: cluster-randomised controlled trial." Lancet 364(9438): 970-979.	Morrison J, Tamang S, Mesko N, Osrin D, Shrestha B, Manandhar M, et al. Women's health groups to improve perinatal care in rural Nepal. BMC Pregnancy Childbirth. 2005;5(6):1-12.	Wade A, Osrin D, Shrestha BP, Sen A, Morrison J, Tumbahangphe KM, et al. Behaviour change in perinatal care practices among rural women exposed to a women's group intervention in Nepal. BMC Pregnancy Childbirth. 2006;6(20):1-10.
Monitoring and evaluation done in participatory way?	Local female enumerators involved in surveillance. After phase one of the intervention, which lasted approximately one year, strategies were implemented and then assessed by group members. Overall study evaluation carried out by the authors alone.	The facilitation team (facilitators, supervisors, and senior facilitation manager) participated in discussions about the intervention and contributed to the participant observation reports used for the analysis. These reports were produced by participant observants- authors (NM and JM, who were also technical advisors to MIRA), who also participated in the design and implementation of the intervention.	See Manandhar et al. 2004 and Morrison et al. 2005.
Duration of intervention	Two years duration presented in this publication. Not clear if the intervention persisted past 2003.	30 months	See Manandhar et al. 2004.
Sustainability	The 10 women's groups developed over a period of nearly a year. No information about length of the implementation and assessment of women's strategies. 95% of the women's groups were still active at the end of the trial.	The majority of the women's groups were still active 30 months after the intervention.	See Manandhar et al. 2004 and Morrison et al. 2005.
Theoretical Framework	Not found in text.	Not found in text.	Not found in text.
Equity considered (PROGRESS)	Not found in text.	Not found in text.	Not found in text.
Cost considerations	The cost per newborn life saved was US$3442 ($4397 including health service strengthening costs) and per life year saved US$111 ($142 including health-service strengthening costs).	See Manandhar et al. 2004.	Not found in text.

Nepal: Makwanpur women’s groups			
Characteristics	Manandhar, D. S., D. Osrin, et al. (2004). "Effect of a participatory intervention with women' groups on birth outcomes in Nepal: cluster-randomised controlled trial." Lancet 364(9438): 970-979.	Morrison J, Tamang S, Mesko N, Osrin D, Shrestha B, Manandhar M, et al. Women's health groups to improve perinatal care in rural Nepal. BMC Pregnancy Childbirth. 2005;5(6):1-12.	Wade A, Osrin D, Shrestha BP, Sen A, Morrison J, Tumbahangphe KM, et al. Behaviour change in perinatal care practices among rural women exposed to a women's group intervention in Nepal. BMC Pregnancy Childbirth. 2006;6(20):1-10.
Study design	Low risk of bias. Cluster randomized controlled trial. 12 matched pairs of village development committees, including 28,931 individual participants who were married women of reproductive age.	See Manandhar et al. 2004 . This paper describes and analyses the implementation of the first stage of the participatory intervention.	See Manandhar et al. 2004. Paper reports a sub-group analysis of women who experienced pregnancy before the intervention and who had another pregnancy during the study period.
Data collection	Baseline data collected from each potential member of the cohort from March - July 2001. Baseline service audit also completed. Menstrual status of individual participants recorded monthly by local female enumerator and structured interviews with women determined to be pregnant were undertaken at 7 months gestation and at 1 month postpartum.	Participant observation by technical advisors (anthropologists) were reported in their monthly reports, which were discussed with and added to by the facilitation team (facilitators, supervisors and senior facilitation manager).	Baseline data collected via structured questionnaire interview of all eligible participants (women who had been pregnant) to identify perinatal practices. At endline, participants who had another pregnancy during study period interviewed with same questionnaire to identify changes in perinatal care practices. 5,400 women eligible for inclusion.
Participants	14,844 married women of reproductive age in the intervention clusters.	The 111 women's groups that emerged in the intervention.	5,400 women who experienced pregnancy before the intervention and who had another pregnancy during the study period.
Outcomes	Primary: neonatal mortality rate. Secondary: stillbirths and maternal deaths, uptake of antenatal and childbirth services, home-care practices at childbirth and postpartum, infant morbidity, and health-care seeking	Analysed and described the development of women's groups, considering factors characteristic of successful groups, the group meetings, and the strategies developed.	Primary Outcomes: attendance at antenatal care; use of a boiled blade to cut the cord; appropriate dressing of the cord; and not discarding colostrum.

Nepal: Makwanpur women's groups			
Characteristics	Manandhar, D. S., D. Osrin, et al. (2004). "Effect of a participatory intervention with women' groups on birth outcomes in Nepal: cluster-randomised controlled trial." Lancet 364(9438): 970-979.	Morrison J, Tamang S, Mesko N, Osrin D, Shrestha B, Manandhar M, et al. Women's health groups to improve perinatal care in rural Nepal. BMC Pregnancy Childbirth. 2005;5(6):1-12.	Wade A, Osrin D, Shrestha BP, Sen A, Morrison J, Tumbahangphe KM, et al. Behaviour change in perinatal care practices among rural women exposed to a women's group intervention in Nepal. BMC Pregnancy Childbirth. 2006;6(20):1-10.
Key Findings	Neonatal mortality rates 30% lower in intervention vs control areas (26.2 vs. 36.9 deaths per 1000 live births, Adj OR: 0.70, 95% CI: 0.53-0.94), and 80% lower maternal mortality ratio (69 vs 341 deaths per 100 000 live births in the intervention arm vs control arm respectively, Adj OR: 0.22, 95% CI: 0.05-0.90). Women in intervention clusters vs control clusters were significantly more likely: to have had antenatal care (Adj OR: 2.82, 95% CI: 1.41-5.62); taken haematinic supplements (Adj OR:1.99, 95% CI:1.14-3.46); given birth in a health facility, with a trained attendant (Adj OR: 3.53, 95% CI: 1.54 8.10) or a government health worker (Adj OR: 3.12, 95% CI:1.62 -6.03); used a clean home childbirth kit (Adj OR: 4.59, 95% CI: 2.83 -7.45) or a boiled blade to cut the umbilical cord (Adj OR: 3.47, 95% CI: 1.39-8.69); for the birth attendant to have washed her hands (Adj OR: 5.5, 95% CI: 2.40-12.6); and to have visited a health facility in the event of maternal (Adj OR: 3.37, 95% CI: 1.78-6.37) or infant (Adj OR: 2.84, 95% CI: 1.65-4.88) illness. Infection-related neonatal deaths were less common in intervention clusters. Members of the women's groups sought more information about perinatal care and raised awareness of perinatal issues outside the groups. Stillbirth rates did not differ between the two groups.	Of the 111 women's groups, 77 developed and designed strategies to address identified problems and 100 continued to meet to discuss perinatal health. The most popular and successfully implemented strategies were: the mother and child health fund, locally produced clean home childbirth kits, management and production of stretchers, and awareness-raising through video shows. Gaps in local knowledge were identified during the planning stages which led to a process of participatory learning being initiated. Authors discuss factors influencing success of groups, reporting that they found 'no specific formula', however, support of husbands, local political organisations, and local health staff seemed important. Authors also discussed some of the challenges emerging from the participatory process, in particular the balance between being directive, particularly regarding health knowledge and education, vs participatory.	Improvements among women not following good practice at baseline in intervention group vs. control in all four indicators. Women not following good practice at baseline were more likely to show a positive improvement if they lived in an intervention area or received the intervention compared with the control. Also, evidence of a greater improvement in attending antenatal care and not discarding the colostrum for women attending groups compared with. Improved attendance at antenatal care: BETTER/WORSE ratio: 1.77 (95% CI: 1.30, 2.40). Improvement in not discarding the colostrum:. BETTER/WORSE ratio 1.03 (95% CI: 1.01, 1.06).

India: Ekjut. Jharkhand and Orissa, women's groups		
Characteristics	Tripathy, P., N. Nair, et al. (2010). "Effect of a participatory intervention with women's groups on birth outcomes and maternal depression in Jharkhand and Orissa, India: a cluster-randomised controlled trial." The Lancet 375(9721): 1182-1192.	Rath, S., N. Nair, et al. (2010). "Explaining the impact of a women's group led community mobilisation intervention on maternal and newborn health outcomes: The Ekjut trial process evaluation." BMC International Health and Human Rights 10(1): 1-13.
Year (of intervention)	2005 - 2008	2005 - 2008
Study location	Jharkhand and Orissa states, India	Jharkhand and Orissa States, India
Brief description of intervention/study	Women's groups were set up or existing women's groups involved in participatory learning and action cycles. Each group met monthly for 20 meetings. Facilitators typically convened 12 - 14 groups per month. Used discussion, games, and stories to identify and prioritise MNH problems in the community, select relevant strategies to address the problems, implement the strategies, and assess the results. Each group was free to implement its own combination of stratgies. Materials used were adapted from the Makwanpur, Nepal study (see Manandhar et al.).	See Tripathy et al.
Type/level of participation	Women's groups facilitated and supported by a local woman through a participatory learning and action cycle, which involved discussions and collective actions including the identification of maternal and neonatal health problems in the community, planning--with other community members--of practical strategies to address the problems, implementation of strategies, and monitoring of strategies.	See Tripathy et al.
Examines effects of community participation?	Yes	Yes.
Pre-existing context	The intervention was implemented in some existing women's groups set up by the NGO Assistance for Development Action (PRADAN).Existing women's groups in 18 clusters participated in credit and savings activities prior to the intervention.	See Tripathy et al.
Who initated the intervention?	External agents (the authors) who 'hypothesised that a participatory intervention with women's groups could reduce neonatal mortality…improve home-care practices and health-seeking behaviour of pregnant and postnatal women…and…reduce maternal depression'.	See Tripathy et al.

India: Ekjut. Jharkhand and Orissa, women's groups		
Characteristics	Tripathy, P., N. Nair, et al. (2010). "Effect of a participatory intervention with women's groups on birth outcomes and maternal depression in Jharkhand and Orissa, India: a cluster-randomised controlled trial." The Lancet 375(9721): 1182-1192.	Rath, S., N. Nair, et al. (2010). "Explaining the impact of a women's group led community mobilisation intervention on maternal and newborn health outcomes: The Ekjut trial process evaluation." BMC International Health and Human Rights 10(1): 1-13.
Relational aspects (e.g. building community support, transferring leadership to participants etc.)	Authors met and sought permission for the intervention and surveillance from village councils, headmen, and representatives from panchayats (elected representatives for basic governance). Other community members who were not regular attendants were also encouraged to participate in the women's group discussions. Also, in all clusters (control and intervention), health committees formed to express opinions about the design and management of local health services.	Critical consciousness' among women's group members enabled them to spread awareness to the wider community, 'as evidenced in group members' support to local village health committees and their involvement of community health workers in discussions about entitlements to health services' (p.9). The presence of frontline government staff, ASHAs, Anganwadi workers, and auxillary nurse midwives at meetings meant that different community members, healthcare workers, and decision-makers have an increased awareness of maternal and neonatal health initiatives in their communities (p.9). The presence of auxillary nurse midwives and Anganwadi workers at meetings reportedly increased their accountabilty to community members.
Symbolic aspects (e.g. addressing women's status, types of social processes mobilised)	At women's meetings participants discussed the difficulties mothers encounter in the community and devised strategies to collectively address them. The authors report that women improved problem-solving skills through participating in the meetings. The authors call for the need to evaluate the outcomes of delivering the intervention in partnership with government and NGOs.	The intervention actively targeted marginalized and pregnant women.
Material aspects (e.g. efforts to achieve sustained funding/symbolic support, enhancing access to material resources, seeking ways for participants to put their new skills into practice elsewhere)	Facilitators received seven-day residential training and fortnightly support from district coordinators. Knowledge about clean childbirth practices and care-seeking enabled local women to address maternal and neonatal health.	Cluster-level meetings allowed women's groups to share problems and strategies with the broader community. As a result, community members, including men, offered to help implement the groups' strategies (p.9). A considerable percentage (over 37%) of home births are attended by family members. These births are likely managed 'using information and skills from the meetings' (p.10). Participation in groups and cluster meetings allowed for participants to become health advocates in their communities and homes, providing assistance to pregnant women (e.g. members recalled providing assistance to 3822 pregnant women during the study period) (p.10).

India: Ekjut. Jharkhand and Orissa, women’s groups		
Characteristics	Tripathy, P., N. Nair, et al. (2010). "Effect of a participatory intervention with women's groups on birth outcomes and maternal depression in Jharkhand and Orissa, India: a cluster-randomised controlled trial." The Lancet 375(9721): 1182-1192.	Rath, S., N. Nair, et al. (2010). "Explaining the impact of a women's group led community mobilisation intervention on maternal and newborn health outcomes: The Ekjut trial process evaluation." BMC International Health and Human Rights 10(1): 1-13.
Leadership, planning and management	Women's groups chose which set of strategies they wanted to implement. The group facilitation materials used to guide the meetings were adapted by the intervention team from the Makwanpur, Nepal study (Manandhar et al. 2004). Health committee members developed knowledge about the government health system and participated in the formation of village health committees as part of the National Rural Health Mission programme.	See Tripathy et al.
External support?	The trial was funded by Health Foundation, UK Department for International Development, Wellcome Trust, and the Big Lottery Fund (UK).	See Tripathy et al.
Monitoring and evaluation done in participatory way?	Group members assessed the results of the strategies that they had implemented. Overall monitoring of the intervention conducted by the authors.	See Tripathy et al.
Duration of intervention	Variable, up to 20 months. The trial was planned for three years.	See Tripathy et al.
Sustainability	The authors state that the intervention costs are lower than those of other primary care interventions, but to manage facilitators, a training and support structure is needed.	Not found in text.
Theoretical Framework	Not specified (though used participatory learning and action cycles). See also Rath et al.	Freirean approach: development of 'critical consciousness'.
Equity considered (PROGRESS)	Women in intervention clusters were poorer and more disadvantaged than those in control clusters.	Ekujut targeted areas predominantly inhabited by tribal people with no or few land holdings, low literacy, and many living below the poverty line. Groups also succesfully targeted pregnant women. See also Tripathy et al.
Cost considerations	Authors report that the incremental cost of women's group intervention (2007 prices) was US$910 per newborn life saved, increasing to $1308 when health service strengthening activities were included. Incremental cost per life-year saved was $33 for the women's group intervention ($48 including health service strengthening activities) (p.1189).	See Tripathy et al.

India: Ekjut. Jharkhand and Orissa, women’s groups		
Characteristics	Tripathy, P., N. Nair, et al. (2010). "Effect of a participatory intervention with women's groups on birth outcomes and maternal depression in Jharkhand and Orissa, India: a cluster-randomised controlled trial." The Lancet 375(9721): 1182-1192.	Rath, S., N. Nair, et al. (2010). "Explaining the impact of a women's group led community mobilisation intervention on maternal and newborn health outcomes: The Ekjut trial process evaluation." BMC International Health and Human Rights 10(1): 1-13.
Study design	Low risk of bias. Cluster-randomised controlled trial. 18 clusters (estimated population of 228 186) from a total of 36 clusters in Jharkhand and Orissa were assigned to the intervention or control arms using stratified randomisation. A total of 244 women's groups emerged in the intervention clusters. In intervention clusters, a facilitator met with 12 - 14 groups every month to go through the participatory action cycles and participatory learning. Intent to treat analysis.	This study describes the context, content, and implementation of the Jharkhand and Orissa women's groups intervention. For intervention study design, see Tripathy et al.
Data collection	Surveillance by key informants in each of the three districts (from the Jharkhand and Orissa states where the study took place) identified all births and maternal and newborn deaths within their jurisdiction. Monthly, the key informant met with an interviewer who verified the births/deaths. Births were followed up approximately six weeks later with a structured questionnaire about sociodemographic characteristics, pregnancy, childbirth, and the postnatal period. Deaths were followed-up with verbal autopsies.	Data were collected through the review of intervention documents, qualitative structured discussions with women's group and non-group members, meeting observation, and statistical analysis of program records.
Participants	Women aged 15-49, residing in the project area, and having given birth during the study.	See Tripathy et al.
Outcomes	Primary: Neonatal mortality rate and maternal depression scores. Secondary: stillbirths; maternal and perinatal deaths; uptake of antenatal care and childbirth services; home care practices during and after childbirth; and healthcare-seeking behaviour.	Qualitative analysis.

India: Ekjut. Jharkhand and Orissa, women's groups		
Characteristics	Tripathy, P., N. Nair, et al. (2010). "Effect of a participatory intervention with women's groups on birth outcomes and maternal depression in Jharkhand and Orissa, India: a cluster-randomised controlled trial." The Lancet 375(9721): 1182-1192.	Rath, S., N. Nair, et al. (2010). "Explaining the impact of a women's group led community mobilisation intervention on maternal and newborn health outcomes: The Ekjut trial process evaluation." BMC International Health and Human Rights 10(1): 1-13.
Key Findings	NMR was 32% lower in intervention clusters compared with control clusters (Adj OR:0.68, 95% CI: 0.59-0.78) during the three years overall and 45% lower in years two and three (Adj OR: 0.55, 95% CI: 0.46-0.66) [adjusted for clustering, stratification, and baseline differences]. No significant effect on maternal depression overall, but reduction in moderate depression by 57% in year three (Adj OR: 0.43, 95%CI: 0.23-0.80) [adjusted for clustering, stratification, maternal education, tribe affiliation, and household assets]. There were improvements in home care practices in intervention clusters (e.g. use of safe childbirth kits).	Six key factors influenced implementation and impact: acceptability among communities; use of a participatory approach to develop knowledge, skills, and a 'critical consciousness'; community involvement beyond the women's groups; a focus on marginalized communities; active recruitment of newly pregnant women; and high population coverage (p.1,12). The authors suggest that the participatory approach was key to its success. The use of stories by the women was entirely participatory and played an important role in educating participants and helping local facilitators familiarize themselves with preventative strategies for common problems (p.8). The authors also suggest community mobilisation may have been the catalyst for developing 'critical consciousness' among group members and the wider community (p.9)

	Mapedir maternal death audits
Characteristics	UNICEF (2008). Maternal and Perinatal Death Inquiry and Response: Empowering communities to avert maternal deaths in India. New Delhi, UNICEF.
Year (of intervention)	2005-2008
Study location	India - various states
Brief description of intervention/study	Maternal and Perinatal Death Inquiry and Response (MAPEDIR). 'Typically, medical records capture only the immediate, biological causes of maternal deaths. The personal, familial, sociocultural, economic and environmental factors contributing to these deaths are left out. MAPEDIR seeks to restore and record these missing links' (p.4). Data are collected using verbal autopsy in the community. Communities are consulted and involved at all stages of the intervention, including devising and implementing strategies to reduce maternal mortality based on the data collected.
Type/level of participation	The intervention supported evidence-based decision-making and advocacy by and with the community, thus nurturing community participation and community empowerment (p.15). However, community members were largely recipients of the intervention. Where the social and political environment was favourable, community participation increased and community members were mobilised to create local maternal health interventions based on the data from the maternal health audits.
Examines effects of community participation?	Community participation in the design of/support of local interventions in response to maternal audit data was a desired outome of the intervention, and where it occurred, it was reported.
Pre-existing context	States were selected due to their higher-than-average maternal mortalities. Prominent tribal populations in some.
Who initated the intervention?	Initiated by UNICEF as an extended component of the Reproductive and Child Health Program with the Government of India.
Relational aspects (e.g. building community support, transferring leadership to participants etc.)	Community members engaged through the involvement of grassroots structures (E.g. Panchayati Raj institutions, village health committees, women's self-help groups), and sensitising them to maternal health issues (p.25). Sharing of the local maternal audit data is done so in such a way as to promote the development of effective local interventions and advocacy by and with the community (p.25). The authors suggest that participation in such meetings is empowering, both for healthcare-givers and seekers (p.41). Additionally, one of the outcomes of MAPEDIR enquiries may be to strengthen linkages with other services e.g. transport providers; arranging systems for emergency transport is reported as one of the key outcomes in many areas where MAPEDIR has been used.
Symbolic aspects (e.g. addressing women's status, types of social processes mobilised)	Interviewers received orientation training in maternal death, and through conducting the audits, gained insight into some of the social and non-medical determinants of health ('I knew the medical causes of maternal mortality in my area...but after my training in MAPEDIR, I also came to know about the non-medical reasons for death: the three delays.' p.56).

	Mapedir maternal death audits
Characteristics	UNICEF (2008). Maternal and Perinatal Death Inquiry and Response: Empowering communities to avert maternal deaths in India. New Delhi, UNICEF.
Material aspects (e.g. efforts to achieve sustained funding/symbolic support, enhancing access to material resources, seeking ways for participants to put their new skills into practice elsewhere)	Mobilisation of funds and restructuring of health systems to improve access to services. (E.g. In response to MAPEDIR maternal death audit data, where local leadership was directly engaged, local village council funds have been used for emergency referral/transport systems (p.41/42)). Authors report that in West Bengal, state action was effectively stimulated and the system strengthened, resulting in efforts being made to improve health infrastructure including "free for all" maternity beds and a cashless (voucher-based) referral and transport system (p.43).
Leadership, planning and management	Intervention was entirely led, planned, and managed externally, although the sharing of audit data findings was sometimes taken up by local NGOs. Design of local interventions, when it occurred, relied on community organizations for planning and implementation.
External support?	Several institutions and groups involved: the Government of India, State Governments, District Administrations, medical faculties of Indian universities, the Johns Hopkins Bloomberg School of Public Health, WHO, UNFPA and UNICEF (p.5).
Monitoring and evaluation done in participatory way?	No. Data compiled and analysed at the district-level with technical assistance from UNICEF.
Duration of intervention	Varied in different locations.
Sustainability	Efforts were made to involve local administration in the process to ensuring scale-up and sustainability (p.18)
Theoretical Framework	Used 'three delays' framework to structure enquiry.
Equity considered (PROGRESS)	MAPEDIR exposed inequities in maternal death by documenting causes related to poverty, low status of woman who died, etc.
Cost considerations	Not found in text.
Study design	Narrative report of implementation of MAPEDIR in different locations: series of primarily qualitative case studies
Data collection	Data from the maternal death audits was collected and analysed continuously throughout the intervention. Collection was by trained local auxillary nurse midwives (ANMs), ANM supervisors, lady health visitors, NGO members, and Integrated Child Development Services supervisors (p.16).
Participants	Communities in different areas of India
Outcomes	Awareness of causes of maternal death that could be tackled at community level. 'The ultimate goal of MAPEDIR is to reduce maternal deaths through communities themselves taking remedial measures to address gaps at the local level, and officials taking corrective measures in the health system' (p.25).

	Mapedir maternal death audits
Characteristics	UNICEF (2008). Maternal and Perinatal Death Inquiry and Response: Empowering communities to avert maternal deaths in India. New Delhi, UNICEF.
Key Findings	Authors report greater community awareness of factors leading to maternal deaths, and relevance of birth preparedness and complication readiness. They also report actions taken as a result e.g. contacting and demanding service from the healthcare delivery system, or involving members of the general community in prevention of maternal death. Example: Dholpur (Rajasthan), village-level transporters have become part of the movement to reduce maternal deaths. In Orissa, increases in childbirth in institutions from 23% to 39% are attributed to the intervention (p.65, but note that there is no control group). In Dhanukapura, a fleet of vehicles was set up to provide transport in cases of obstetric emergency.

Additional studies (high risk of bias)
Foundation for Research in Health Systems. (2004). Community involvement in reproductive health: findings from research in Karnataka, India. USAID. Bangalore, India.
Fofana, P., Samai, O., Kebbie, A., Sengeh, P. (1997). "Promoting the use of obstetric services through community loan funds, Bo, Sierra Leone. The Bo PMM Team" International Journal of Gynecology and Obstetrics 59 (SUPPL. 2): S225-S230.
Turan, J. M., Tesfagiorghis,M, Polan, M.L. (2011). "Evaluation of a Community Intervention for Promotion of Safe Motherhood in Eritrea" Journal of Midwifery and Women's Health 56 (1): 8-17.
