# Supplementary figures and images for: Effects of Community Participation on Improving Uptake of Skilled Care for Maternal and Newborn Health: A Systematic Review
Source: PLoS One. 2013 Feb 4;8(2):e55012. doi: 10.1371/journal.pone.0055012 (PMC3563661; doi:10.1371/journal.pone.0055012)

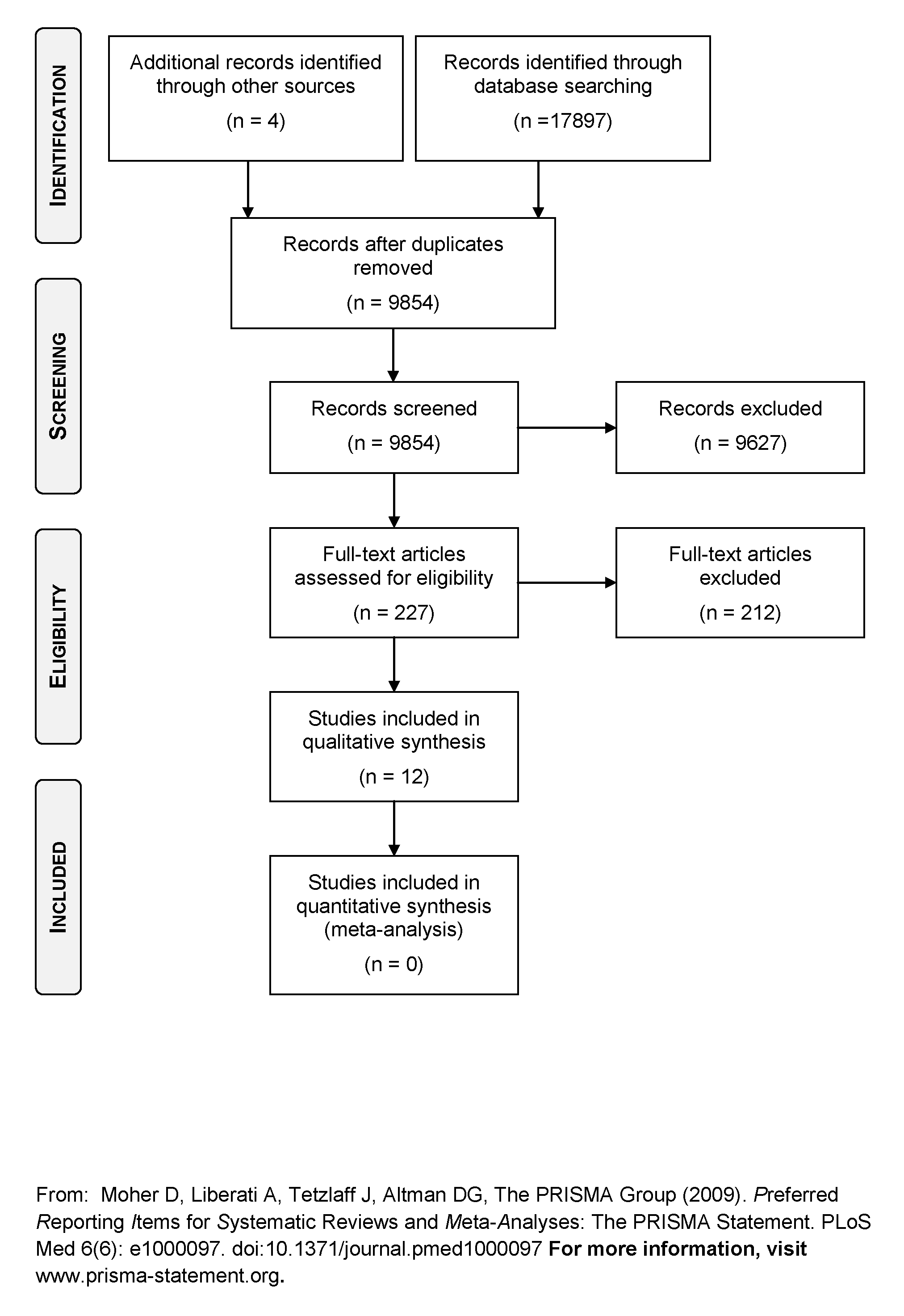

Supplement: Checklist S1 — PRISMA flow diagram. (TIF) [file pone.0055012.s002.tif]
